# Phylogenomic mixture models outperform homogeneous and partitioned models

**DOI:** 10.1093/molbev/msag090

**Published:** 2026-04-09

**Authors:** Davide Pisani, Mattia Giacomelli, Gergely J Szöllősi, Maria Eleonora Rossi, Marc Domènech, Jesus Lozano-Fernandez

**Affiliations:** Bristol Palaeobiology Group, School of Biological Sciences, University of Bristol, Bristol BS8 1TH, UK; Department de Genètica, Microbiologia i Estadística, Facultat de Biologia, Universitat de Barcelona, Barcelona 08028, Spain; Institut de Recerca de la Biodiversitat (IRBio), Universitat de Barcelona, Barcelona, Spain; Model-Based Evolutionary Genomics Unit, Okinawa Institute of Science and Technology Graduate University, Okinawa, Japan; HUN-REN Centre for Ecological Research, Institute of Evolution, Budapest 1121, Hungary; Centre for Chromosome Biology, School of Biological and Chemical Sciences, University of Galway, Galway H91 W2TY, Ireland; Department de Genètica, Microbiologia i Estadística, Facultat de Biologia, Universitat de Barcelona, Barcelona 08028, Spain; Institut de Recerca de la Biodiversitat (IRBio), Universitat de Barcelona, Barcelona, Spain; Department de Genètica, Microbiologia i Estadística, Facultat de Biologia, Universitat de Barcelona, Barcelona 08028, Spain

**Keywords:** compositional heterogeneity, mixture models, simulations, model fit, phylogenetic accuracy, long branch attraction

## Abstract

Significant advances have been made in resolving the tree of life, but many nodes remain debated. The last two decades saw the emergence of mixture models, which proved particularly useful to account for across-site compositional heterogeneity, and played a central role to improve our understanding of difficult phylogenetic problems. However, some scholars have remained skeptical of their use. Here, we perform a large simulation study comparing mixture models accounting for across-site compositional heterogeneity, across-site compositionally homogeneous models and partitioned models. We show that the tested mixture models fit across-site compositionally heterogeneous datasets best and achieve greater accuracy. Of the tested models, CAT-GTR, an infinite mixture model combining a general time reversible—GTR—matrix with a mixture of site-frequency profiles (i.e. categories—CAT—or components) characterized by different amino acid frequency vectors, maximizes accuracy and fit. Mixture models, and particularly CAT-GTR, perform well also with across-site compositionally homogeneous datasets, where the use of a mixture of site-frequency profiles is not necessary. We show that this is because with homogeneous data these models converge to appropriate compositionally homogeneous models, avoiding overparametrization. Our results dissipate doubts about the utility of models accounting for compositional heterogeneity across sites and identify CAT-GTR as one of the most flexible models in the phylogenomic arsenal.

## Introduction

Resolving the tree of life remains a main goal of evolutionary biology, and while genome-scale datasets have increased our understanding of its backbone, many nodes remain hard to resolve. Recently debated examples cover a diversity of timescales ranging from the root of eukaryotes (Al Jewari and Baldauf 2023; Williamson et al. 2025), to that of animals (Dunn et al. 2008; Philippe et al. 2009; Pisani et al. 2015; Whelan et al. 2015; Feuda et al. 2017; Pett et al. 2019; Li et al. 2021; Juravel et al. 2023; Schultz et al. 2023; Copley 2025), and even the root of more recently diverged groups such as the ants (Romiguier et al. 2022; Cai 2024; Boudinot and Lieberman 2025; Tao et al. 2026). These debates might involve the interpretation of results from different data types, such as the debate on the root of Metazoa (Ryan et al. 2013; Pisani et al. 2015; Pett et al. 2019; Juravel et al. 2023; Schultz et al. 2023; Copley 2025). However, when sequence data (amino acid –AA– or nucleotide sequences; here we focus on AA) are considered, disagreement usually revolves around how the substitution process is modeled (see for example, Philippe et al. 2011; Pisani et al. 2015; Feuda et al. 2017; Whelan and Halanych 2017; Wang et al. 2019; Li et al. 2021; Susko and Roger 2021; Giacomelli et al. 2022; Szantho et al. 2023; Banos et al. 2024a).

To understand the relevance of modeling in phylogenomics, it is useful to start from an exemplar model that was used in early studies of molecular evolution: the Poisson process, see Nei and Kumar (2000) for an introduction. A Poisson process assumes uniform AA exchangeabilities, equal AA frequencies, and a homogeneous substitution rate across sites. It is sufficient to eyeball a protein alignment to conclude that AA substitutions do not follow a Poisson process, as blocks of sites differing in the number and chemical properties of the AA that they display can be easily identified, which is inconsistent with the uniformity assumptions of the Poisson process. Real evolutionary processes are not uniform, and properties such as exchangeability rates, AA frequencies, and rates of substitution vary across both sites and lineages. Many models have been developed to relax these assumptions. For example, general time reversible (GTR) matrices, including empirical GTRs such as the Whelan and Goldman (WAG) matrix (Whelan and Goldman 2001), were introduced to relax the assumption of homogeneity of the AA replacement rate (Lanave et al. 1984; Yang 1994a).

Across-site compositional heterogeneity (hereafter also simply referred to as heterogeneity—as our study does not concern other forms of heterogeneity) is pervasive in sequence alignments (Lartillot et al. 2007; Wang et al. 2019; Szantho et al. 2023). This form of heterogeneity is caused by functional constraints: sites that play fundamental structural or functional roles are under purifying selection, accepting fewer substitutions from a restricted alphabet composed of a subset of the 20 amino acids (Lartillot et al. 2007; Szantho et al. 2023). For example, a site that must maintain a hydrophobic profile is more likely to accept substitutions between hydrophobic AAs than between hydrophobic and polar AAs. At stationarity, these sites are expected to have different AA equilibrium frequencies, hence the observation of sites of different composition in AA alignments (see above). To account for site-specific differences in alignments (a phenomenon that is also referred to as pattern heterogeneity), Lartillot and Philippe (2004) introduced the Categories (CAT)-based models: CAT-Poisson (applying a uniform AA-replacement rate) and CAT-GTR, which models AA-replacement rates with a GTR matrix. While CAT-based models (i.e. CAT-Poisson and CAT-GTR) have been in existence for more than 20 years, they surprisingly remain poorly understood. For example, Boudinot and Lieberman (2025) claimed that these models do not account for compositional heterogeneity because they are rate models. CAT-based models are indeed rate models, but they assume that the sites in an alignment do not evolve under the same processes (Lartillot and Philippe 2004). To account for that, CAT-based models use multiple site-frequency profiles (see below for details) which are characterized by different AA composition vectors, therefore accounting for the compositional heterogeneity observed across sites.

To understand CAT-based models, we first introduce the Dirichlet processes. Dirichlet processes define infinite mixture models and are frequently explained using the analogy of the Chinese Restaurant Process (Gershman and Blei 2012), in which customers (in our case sites) enter a restaurant and choose to sit either at an existing table (in our case joining an existing site-partition) with probability proportional to its occupancy, or at a new table (forming a new partition). This process defines a flexible, potentially unbounded number of clusters/partitions. When using a Dirichlet process to explain an alignment, we assume that sites are drawn from a population consisting of a potentially unbounded number of site classes, each characterized by a different AA frequency profile. The Dirichlet process therefore clusters the sites in the alignment across a set of partitions (categories or components), the number of which is not known a priori, and is expected to reflect the distribution of site classes from which the sites in the alignment were pooled. Each site category is characterized by (1) a frequency vector describing the AA equilibrium frequencies observed across the sites assigned to it and (2) a weight corresponding to the proportion of sites it describes.

Practical implementations of CAT-based models use truncated, rather than full (infinite), Dirichlet processes. Accordingly, the distribution of categories used by CAT-based models is not infinite; it is cut at a large but finite number that is set in Phylobayes (Lartillot et al. 2013) using the -*nmax* flag (default 1,000). When CAT-based models are used, the number of categories depends on the heterogeneity of the alignment. However, the number of categories is always finite, and their weight and composition are inferred from the data. CAT-based models are non-parametric in the Bayesian sense, because the number of categories they use (and hence their number of parameters—each category adds its own AA frequency profile and its weight) is not fixed before the analysis, it is explored during model fit.

An intuitive way to think about this is as a “menu” that can expand or contract. You start with a set of “typical” amino-acid “recipes”, with each “recipe” being a different amino-acid palette (a different equilibrium frequency vector). Recipes will have different levels of popularity, as sites under similar constraints tend to share recipes, and you only add a new recipe if some sites exist that have a composition pattern that none of the existing recipes can capture. The number of recipes necessary to describe the data is explored, and as the Bayesian analysis progresses, it can increase or decrease. The non-parametric nature of CAT-based models sets them apart from parametric models such as GTR, where the number of parameters might be large (an AA GTR matrix has 189 rate parameters) but is fixed and does not change when different datasets are used, or as the Markov chain Monte Carlo (MCMC) progresses.

The number of categories used by CAT-based models varies and can theoretically be extremely large. However, sites sharing similar biochemical constraints will have similar composition and share the same profile. Accordingly, the number of categories used in CAT-based analyses is always (at least in our experience) much smaller than the theoretical maximum, which is always equal to the length of the alignment (representing the limiting case in which every site is partitioned into its own category). The number of components used in empirical studies is further controlled by the limit imposed by *nmax* (which is generally much lower than the length of modern phylogenomic alignments) and by the Dirichlet process prior itself, which implements an implicit complexity penalty that discourages sites from joining sparsely populated or empty categories: the rich-get-richer assumption (Poux-Médard et al. 2023), mitigating the risk of overfitting (Gershman and Blei 2012). Nevertheless, the fact that CAT-based models can in theory instantiate a seemingly unbounded number of categories has been presented as a concern by authors skeptical of their utility, e.g. Li et al. (2021). While this is a reasonable worry, there is confusion surrounding the possible complexity of CAT-based models. For example, Boudinot et al. (2023) suggested that CAT-based models are expected to have high likelihood because they are theoretically infinitely complex. This reflects a misunderstanding as “infinite” refers to the model's flexibility, the fact that the number of categories is not fixed in advance, rather than to the number of categories actually used.

CAT-based models are partitioned, as they use a mixture of components to describe the data. Each component has a weight representing how much of the alignment it describes, and this defines the category's contribution to the likelihood of the alignment, see also Lartillot (2020). The probabilistic partitioning implemented in CAT-based models therefore implies that poorly populated categories contribute little to the likelihood of the alignment—their contribution being proportional to their weight. This property is important as it protects these models against overparametrization (see below for details), while still allowing a fine modeling of heterogeneity. Furthermore, the use of probabilistic partitioning sets aside CAT-based models from standard partitioned models, e.g. Lanfear et al. (2012), which implement a hard assignment of sites to partitions that are set before the analyses are run. This is because probabilistic partitioning allow assuming ingornace about what sites evolved under different processes, which cannot be done when a hard partitioning of the sites is implemented, see Susko et al. (2026) for a detailed mathematical treatment of this issue.

Following Lartillot and Philippe (2004), a diversity of mixture models accounting for across-site compositional heterogeneity were developed (Le et al. 2008, 2012; Schrempf et al. 2020; Banos et al. 2024b), with the most frequently used ones remaining the CAT-based models themselves and the empirical profile mixture models with fixed numbers of site-frequency categories of Le et al. (2008). The latter are here referred to as CXX (where XX can be 10, 20, …, 60; see e.g. the IQTree2 manual; https://IQTree.github.io/doc/Command-Reference).

Similarly to CAT-based models, CXX models assume that different sites evolve under different processes without specifying which site evolve under which process (Susko et al. 2026). To achieve this, the likelihood of each site is calculated for each component, and then multiplied by the weight of the component itself, allowing to weight the contribution of different components to the likelihood of the alignment based on the proportion of sites they describe. This achieve the same effect of the probabilistic partitioning used by CAT-based models. However, differently from CAT-based models, CXX models are parametric as the number of site-frequency profiles used is fixed. A further difference is that CXX models use precomputed AA frequency vectors and precomputed component weights which might not necessarily describe different superalignments equally well. Nonetheless, CXX models have important areas of application, e.g. in the analysis of single gene alignments, and recent IQTree2 (Minh et al. 2020) implementations increased their flexibility allowing users to infer component weights from the data.

CXX models can be combined with a Poisson process or an amino acid replacement matrix to implement models such as CXX-Poisson, CXX-WAG, or CXX-GTR. In addition, both CXX- and CAT-based models can be combined with a Gamma (+G) distribution (Yang 1994b) to model across-site rate heterogeneity, generating models such as WAG + CXX + G or CAT-GTR + G.

CAT- and CXX-based models have been shown to fit heterogeneous datasets better than across-site compositionally homogeneous models, hereafter referred to as homogeneous models, such as GTR and WAG (Morgan et al. 2013; Feuda et al. 2017; Bujaki and Rodrigue 2022; Cai et al. 2022, 2024; Giacomelli et al. 2022, 2025; Munoz-Gomez et al. 2022; Redmond 2024). However, based on 52 simulated alignments, Whelan and Halanych (2017) concluded that the performance of CAT-based models was not better than that of homogeneous models, with partitioned models, which are heterogeneous across genes but homogeneous within partition, performing as well as CAT-GTR. Wang et al. (2019) challenged the conclusions of Whelan and Halanych (2017), but the results of Whelan and Halanych (2017) continue to be cited to dismiss CAT-based models, e.g. Al Jewari and Baldauf (2023) and Boudinot and Lieberman (2025). Other authors, such as Li et al. (2021) seem convinced of the utility of CXX-based mixture models but remain skeptical of CAT-based models that they suggest use too many sparsely populated categories, potentially overfitting the data. However, the rationale on which the argument of Li et al. (2021) is based is not clear, given that both CAT-based models and CXX models assign weights to components that are proportional to the number of sites they represent (i.e. how well populated they are), and that Banos et al. (2024a) showed that this effectively shields these models from overparametrization, because the smaller the weight of a category is, the smaller its contribution to the likelihood of the alignment.

Cai et al. (2024) performed simulations suggesting that when CAT-GTR is applied to homogeneous datasets it converges to GTR, instantiating models that (after convergence) use a single site-frequency profile in most MCMC generations. Cai et al. (2024) results could constitute an empirical rejection of Li et al. (2021) conclusions, given that, if Li et al. (2021) were correct, CAT-GTR should be expected to overparametrize homogeneous datasets. This is because the use of multiple site-frequency profiles is unnecessary when a GTR matrix is used to account for AA replacement rate heterogeneity, in otherwise homogeneous datasets. However, the simulations of Cai et al. (2024) were limited in scope and number and need to be validated. In addition, given that Whelan and Halanych (2017) and Wang et al. (2019) reached different conclusions, an independent test of the relative performance of homogeneous, heterogeneous, and partitioned models seems necessary to break the impasse.

We completed a large simulation study to test the relative performance of homogeneous, partitioned, and heterogeneous (hereafter also referred to as mixture, or compositionally heterogeneous) models. The latter are represented by CXX-based models and by a CAT-based model (CAT-GTR). We found that the performance of different models depends on the heterogeneity of the data and the lengths of the branches of the target tree. Homogeneous models perform well with homogeneous data, while heterogeneous models perform well with both homogeneous and heterogeneous data. CAT-GTR emerges as the best performing among the model tested. The accuracy of homogeneous and partitioned models is significantly worse than that of CXX and CAT-based models when the data are heterogeneous, rejecting Whelan and Halanych (2017) conclusions. To understand the difference between our results and those of Whelan and Halanych (2017), we tested the heterogeneity of their simulated datasets, finding that their approach failed to incorporate heterogeneity in their simulation. Our results therefore show that the conclusions of Whelan and Halanych (2017) are contingent on the properties of their simulated datasets and do not provide a reliable basis for informing real-world phylogenomic studies.

## Results

### Simulated data and their heterogeneity

#### Datasets simulated in our study

**Figure 1:**
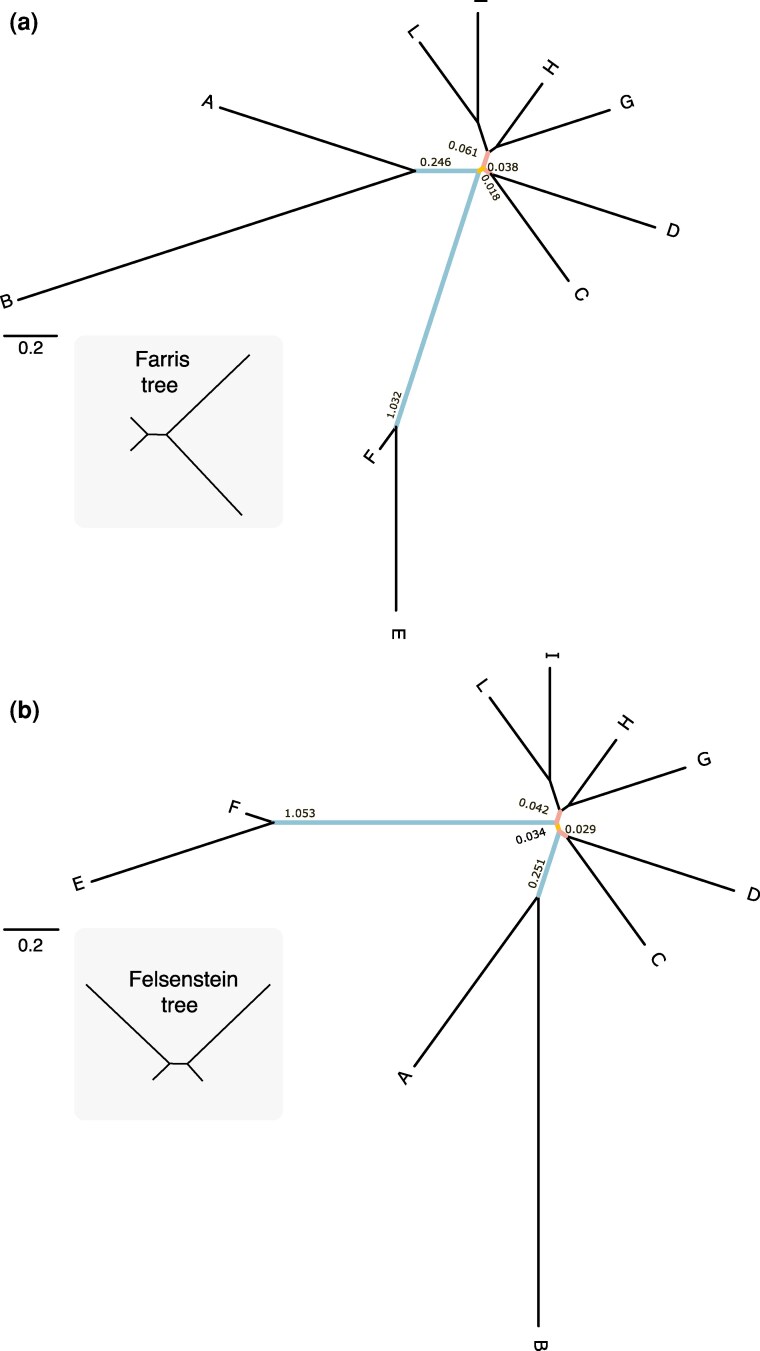
Target trees with branch lengths optimized using real data (BLMF = 1). a) The Farris tree. Farris trees are characterized by having a clade composed of two long-branched lineages separated by a short branch from a clade composed of two short-branched lineages (see Methods and Figures S1–S6). b) The Felsenstein tree. Felsenstein trees are characterized by having two clades, each composed of one long-branched and one short-branched lineage, separated by a short branch (see Methods and Figures S1–S6). Light blue: long-branched lineages. Light yellow: the short-interbranch. Light pink: short-branched lineages. Numbers along the branches are target branch lengths (used in the experiments reported in Fig. 5). The gray boxes present stylized version of the Farris and Felsenstein backbone trees. From these two trees, 6 more target trees were obtained by multiplying the length of the two light-blue branches using BLMF = 0.1, 5, and 10, respectively (see Figures S1–S6) and Methods. These trees are available in Newick format: https://doi.org/10.6084/m9.figshare.29404982.

We performed simulations using two target trees, a “Farris” and a "Felsenstein” tree (Kapli and Telford 2020; Giacomelli et al. 2022), Fig. 1, Figures S1–S6. Together, these two trees represent a well-known phylogenetic problem (Swofford et al. 2001) where, as the lengths of the two long-branched lineages (see Fig. 1) increase (see Figures S4–S6), the probability of inferring the Farris tree increases even when the Felsenstein tree has been used to simulate the data (Felsenstein 1978). We tested the ability of homogeneous and heterogeneous models to recover both trees, with “Small” (7,743 sites and 10 taxa) and “Large” (30,942 sites and 10 taxa) homogeneous and heterogeneous datasets simulated using Elynx (Szantho et al. 2023), see Methods. We tested the ability of the considered models to recover the target trees as the length of the long branches in Fig. 1 was either reduced (Figures S1 and S2) or extended (Figures S3–S6), to monitor the propensity of different models to return trees displaying long branch attraction (LBA) artifacts. Finally, we tested the ability of homogeneous, partitioned, and mixture models at recovering the Felsenstein tree using small (7,740 sites and 10 taxa), concatenated, 5-gene datasets simulated using Phylobayes (Lartillot et al. 2013), see Methods for details. The latter set of analyses was performed to allow a meaningful inclusion of partitioned models in our study, as testing partitioned models on single-partition datasets (i.e. our Elynx datasets) would not have been appropriate. See https://doi.org/10.6084/m9.figshare.29404982 for the target trees, the simulated data, and all the scripts and parameter files used to simulate the data.

We used model adequacy tests implemented using posterior predictive analysis (PPA) in Phylobayes to test whether the across-site compositional heterogeneity of our small and large datasets could be adequately described using GTR. This is a way to make sure that our homogeneous and heterogeneous datasets are truly either homogeneous or heterogeneous, as GTR will fail to adequately describe heterogeneous datasets (Feuda et al. 2017). Our tests compared the average amino acid diversity of our datasets against that of posterior predictive simulated datasets (Feuda et al. 2017; Giacomelli et al. 2022, 2025). A model was considered to fit the data when (−2 < Z-Score < 2). That is, when the amino acid diversity of the tested dataset was within 2 standard deviations from the average amino acid diversity estimated for the posterior predictive simulated datasets, see Methods for details. First, we tested whether GTR could adequately fit the 32-gene superalignment used to parametrize the models implemented in our simulations (Methods for details), observing a PPA-Z-Score_GTR-realdata_ = 11.39. This result confirms the heterogeneity of our model dataset. The small and large (Elynx simulated) homogeneous datasets were adequately modeled by GTR (average PPA-Z-Score_GTR_ = 0.0177 – SD = 0.28 and average PPA-Z-Score_GTR_ = 0.38 – SD = 0.22, respectively), confirming their homogeneity. Differently, the small and large heterogeneous (Elynx simulated) datasets were not adequately modeled by GTR (average PPA-Z-Score_GTR_ = 6.169 – SD = 0.41 and average PPA-Z-Score_GTR_ = 12.19 – SD = 0.81, respectively), confirming their heterogeneity. The (Phylobayes) concatenated 5-gene datasets were not adequately modeled by GTR (PPA-Z-Score_GTR_ = 10.86 – SD = 0.94), confirming their heterogeneity. All these results are reported in Allresults.xls (https://doi.org/10.6084/m9.figshare.29404982).

#### The heterogeneity of Whelan and Halanych (2017) datasets

We tested whether the 52 “heteroGenes” datasets of Whelan and Halanych (2017), i.e. their “heterogeneous” datasets, could be adequately described by GTR. The largest majority (71%) of these datasets were adequately described by GTR, indicating that they were not heterogeneous. Whelan and Halanych (2017) short indel-Seq-Gen (iSG) datasets have an average PPA-Z-Score_GTR_ = 1.24 (SD = 0.829). Only three of these datasets have a PPA-Z-Score_GTR_ > 2, with the most heterogeneous of their iSG datasets (S50_T1_4_heteroGenes) having a PPA-Z-Score_GTR_ = 3.39. Their six long iSG datasets have average PPA-Z-Score_GTR_ = 3.51 (SD = 1.91). This group of datasets includes their most heterogeneous dataset overall, L19_TB_1_heteroGenes (PPA-Z-Score_GTR_ = 6.92). Their short and long INDELible simulated datasets had, respectively, average PPA-Z-Score_GTR_ = 1.12 (SD = 0.79) and average PPA-Z-Score_GTR_ = 2.38 (SD = 1.296). The most heterogeneous INDELible dataset L19_TB_1_heteroGenes had PPA-Z-Score_GTR_ = 4.526. For comparison we tested whether GTR could describe some of their homogeneous datasets, finding no difference in fit. For example, the iSG simulated L19_TB_1 and L19_TB_1_heteroGenes (its heterogeneous counterpart) had PPA-Z-Score_GTR_ = 6.74 and 6.92, only marginally different. The homogeneous INDELible simulated L19_TB_1 and L19_TB_1_heteroGenes had PPA-Z-Score_GTR_ = 4.9 and 4.526. In this case GTR found it marginally harder to fit the “homogeneous” dataset. See Allresults.xls (https://doi.org/10.6084/m9.figshare.29404982).

#### Convergence of Bayesian analyses

Our study compares results of Bayesian (GTR and CAT-GTR) and maximum likelihood analyses, see Methods for justifications. To reduce computational time and emissions, see Methods and Kumar (2022), only one MCMC chain was run for each dataset (10,000 generations with a burnin of 5,000 generations), for all our Bayesian analyses. We tested whether the length to which we ran our chains should be sufficient to achieve convergence for ten small and large datasets. This test was performed under CAT-GTR, which requires more generations to reach convergence. For these 20 datasets, we ran two chains and calculated convergence statistics using *bpcomp* and *tracecomp* (Lartillot et al. 2013). These results, summarized here and presented in full in Allresults.xls (https://doi.org/10.6084/m9.figshare.29404982), show that running the analysis for 10,000 generations (burnin = 5,000) was sufficient for the scope of our analyses: average *maxdiff* = 0.02 (small datasets) and 0.00076 (large datasets); average *effsize* =1,795.65 (small datasets) and 1,589.47 (large datasets) and average *rel_diff* = 0.054 (small datasets) and 0.0611 (large datasets).

### Comparing homogeneous and mixture models on datasets simulated with Elynx

#### Trees with original branch lengths—branch length multiplier factor (BLMF = 1; see Methods)

**Figure 2:**
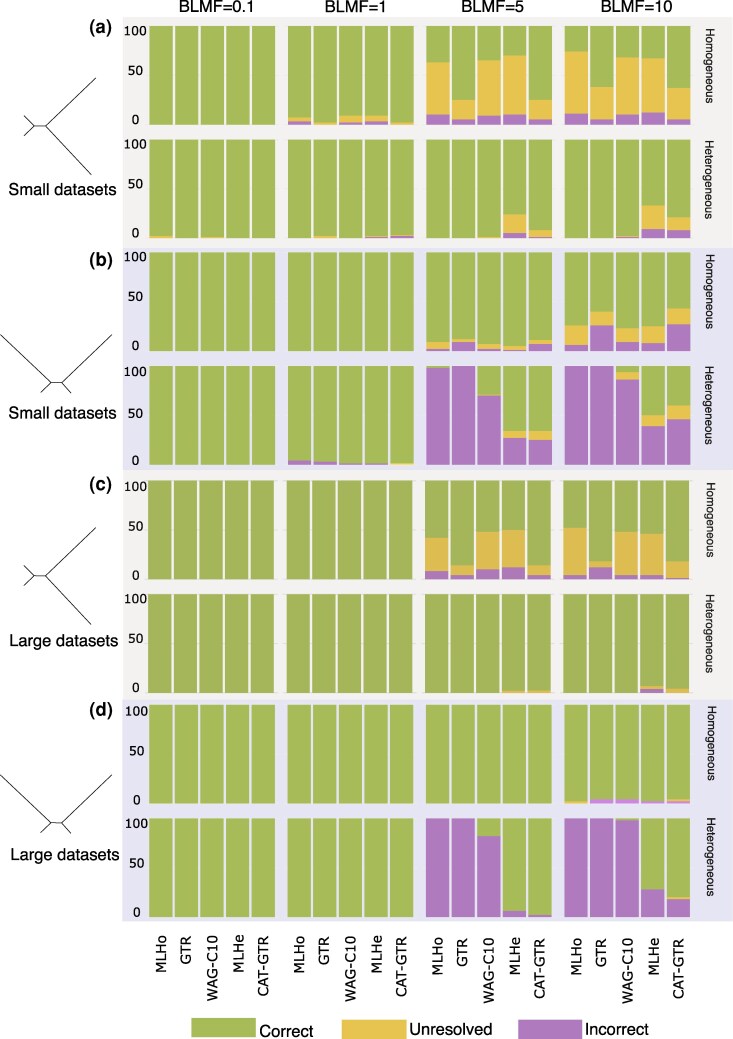
Phylogenetic accuracy: Elynx-simulated datasets. Recovery Rate (RR: the frequency with which the target tree is recovered) represented against the frequency with which unresolved (U) and erroneous/incorrect (Wrong; W) trees are inferred, for the datasets simulated using Elynx. a) small datasets, Target tree = Farris. b) small datasets, Target tree = Felsenstein. c) large datasets, Target tree = Farris. d) large datasets, Target tree = Felsenstein. Green = Correct; Yellow = Unresolved; Purple = Incorrect. We consider unresolved any tree with support lower than 50% (either ultrafast bootstrap or posterior probability) irrespective of its topology. Models are on the X-axis; on the Y-axis, we report percentages for RR, U, and W.

For the Farris tree of Fig. 1a and the homogeneous small datasets, GTR and CAT-GTR are the most successful models achieving a recovery rate (RR—the number of times the correct tree was inferred) of 98% (RR = 98). The two models with the worst RR are the best fitting heterogeneous model with a fixed number of categories (hereafter MLHe), and WAG-C10. In all our analyses of homogeneous datasets, MLHe and WAG-C10 were set as WAG + F + G + CXX (with XX = 10 or 60), and the optimal components' weights were inferred from the data (Methods for details). Both models (MLHe and WAG-C10) had RR = 91, Fig. 2a and Table S1. When the data are heterogeneous, the best fitting homogeneous model identified by IQTree2 (hereafter MLHo) using the BIC (Kalyaanamoorthy et al. 2017), see Methods for details, always recovers the target tree (RR_MLHo_ = 100). The RR of GTR is RR_GTR_ = 98. The RR of WAG-C10 (set as above—Methods for details) is RR_WAG-C10_ = 100. The RR of MLHe is RR_MLHe_ = 98. For the heterogeneous datasets, the MLHe model was identified using the BIC to compare alternative WAG + F + G + CXX models (with XX = 10, 20, …, 60). The optimal components' weights of the selected model were inferred from the data, see Methods for an explanation of the different rationales used to set MLHe with homogeneous and heterogeneous data. The RR of CAT-GTR is RR_CAT-GTR_ = 97.

For the Felsenstein tree and the homogeneous small datasets the RR is maxed for all models (RR = 100), Fig. 2b and Table S1. When the data are compositionally heterogeneous, RR is still high (96 < RR < 99) depending on the model, Fig. 2b and Table S1. MLHo is the model with the worst RR (RR_MLHo_ = 96). The three heterogeneous models WAG-C10, MLHe, and CAT-GTR performed best and had the same RR (RR = 99). When analyzing the large datasets RR = 100 for both trees, under all models with all datasets, Fig. 2c and d and Table S2.

#### Trees with short branch lengths—BLMF = 0.1

When the lengths of the long-branched lineages are reduced by one order of magnitude (see Methods), all models have very high RRs (98 < RR < 100), across all experimental conditions, Fig. 2a–d and Tables S1 and S2.

#### Trees with long branch lengths—BLMF = 5

When the two long-branched lineages are extended 5 times, the RR for the two target trees changes depending on the model used, and on whether the data are homogeneous or not. For the Farris tree (small homogeneous datasets) MLHo, WAG-C10 and MLHe perform poorly (RR_MLHo_ = 37, RR_WAG-C10_ = 30 and RR_MLHe_ = 30). GTR and CAT-GTR have the same, relatively good RR (RR = 75—Fig. 2a and Table S1). With the large homogeneous datasets the RRs of MLHo, WAG-C10 and MLHe remain poor (RR_MLHo_ = 58, RR_WAG-C10_ = 52, RR_MLHe_ = 50), while GTR and CAT-GTR improve (RR_GTR_ = 90 and RR_CAT-GTR_ = 84), Fig. 2c and Table S2. For the Felsenstein tree (small homogeneous datasets) RR_MLHo_ = 91, RR_WAG-C10_ = 93, and RR_MLHe_ = 95 (Fig. 2b and Table S1). GTR and CAT-GTR had lower RRs (RR_GTR_ = 88 and RR_CAT-GTR_ = 89). With the large homogeneous datasets, all models had RR = 100, Fig. 2d and Table S2.

When the data are heterogeneous, the picture changes drastically. For the Farris tree (small datasets), RR_MLHo_ = 100, RR_GTR_ = 100 and RR_WAG-C10_ = 99—Fig. 2a and Table S1. MLHe has the worst RR (RR_MLHe_ = 76). CAT-GTR performs well even if not as well as MLHo, GTR and WAG-C10 (RR_CAT-GTR_ = 92—Fig. 2a and Table S1). With the large datasets, the RRs of MLHo, GTR, and WAG-C10 are maximal (RR = 100). The RR of MLHe and CAT-GTR increases (RR_MLHe_ = 98 and RR_CAT-GTR_ = 94), Fig. 2c and Table S2. For the Felsenstein tree (small datasets), RR_MLHo_ = 2, RR_GTR_ = 0 and RR_WAG-C10_ = 29, Fig. 2b and Table S1. MLHe and CAT-GTR perform much better (RR = 66). With the large datasets RR_MLHo_ = 0, RR_GTR_ = 0 and RR_WAG-C10_ = 18, Fig. 2d and Table S2. MLHe and CAT-GTR improve their RR significantly (RR_MLHe_ = 94, RR_CAT-GTR_ = 98), performing significantly better than the homogeneous models and of WAG-C10 (Fig. 2d and Table S2).

#### Trees with very long branch lengths—BLMF = 10

When the long-branched lineages are extended by one order of magnitude, model-specific differences become very evident. With the small homogeneous datasets, both target trees appear difficult to identify. For the Farris tree, GTR and CAT-GTR are the models with the highest RR (RR_GTR_ = 62 and RR_CAT-GTR_ = 63). MLHo has the worst RR (RR = 26), Fig. 2a and Table S1. For the Felsenstein tree GTR and CAT-GTR have poor RRs (RR_GTR_ = 60 and RR_CAT-GTR_ = 57). The highest RR is achieved by WAG-C10 (RR_WAG-C10_ = 77), followed by MLHe (RR_MLHe_ = 75) and MLHo (RR_MLHo_ = 74), Fig. 2b and Table S1. Using the large datasets improves RRs for all models. For the Farris tree, GTR and CAT-GTR have the best RR (RR = 82), Fig. 2c and Table S2. The other models have RR_MLHo_ = 48, RR_WAG-C10_ = 52, and RR_MLHe_ = 54, Fig. 2c and Table S2. For the Felsenstein tree, all models perform similarly well. MLHo and MLHe have RR = 98, with all the other models having RR = 96, Fig. 2d and Table S2.

With heterogeneous datasets, the RR of different models changes drastically. With the Farris tree (small datasets) RR_MLHo_ = 100, RR_GTR_ = 100, and RR_WAG-C10_ = 98, Fig. 2a and Table S1. In these analyses, RR_CAT-GTR_ = 79 and RR_MLHe_ = 64. With the large datasets, the RR of MLHo, GTR, and WAG-C10 is maximal (RR = 100). The RR of CAT-GTR and MLHe also improves significantly (RR_CAT-GTR_ = 92 and RR_MLHe_ = 94), Fig. 2c and Table S2. For the Felsenstein tree (small datasets) RR_MLHo_ = 0, RR_GTR_ = 0, and RR_WAG-C10_ = 7, Fig. 2b and Table S1. MLHe has the highest RR (RR_MLHe_ = 50) while RR_CAT-GTR_ = 40, Fig. 2b and Table S1. For the large datasets, MLHo, GTR and WAG-C10 continue to have very poor RRs (0 < RR < 2), Fig. 2d and Table S2. MLHe and CAT-GTR improve significantly with CAT-GTR having the best RR (RR_CAT-GTR_ = 80 and RR_MLHe_ = 72), Fig. 2d and Table S2.

#### Comparing across-site compositionally homogeneous, heterogeneous, and partitioned models

**Figure 3:**
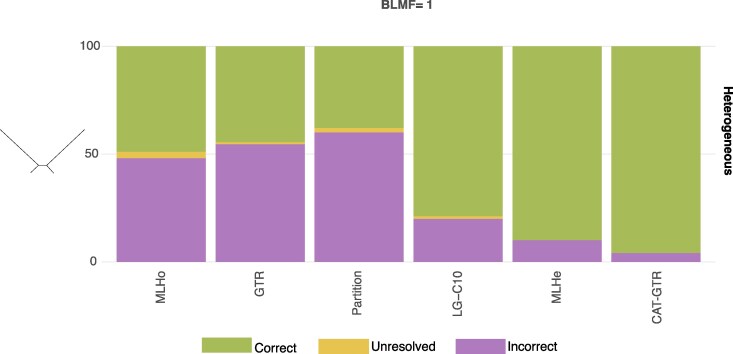
Phylogenetic accuracy: Phylobayes-simulated datasets. Recovery Rate (RR: the frequency with which the target tree is recovered) represented against the frequency with which unresolved (U) and erroneous/incorrect (Wrong; W) trees are recovered for the heterogeneous 5-gene datasets simulated using Phylobayes. Target tree = Felsenstein, BLMF = 1. Green = Correct; Yellow = Unresolved; Purple = Incorrect. We consider unresolved any tree with support lower than 50% (either ultrafast bootstrap or posterior probability) irrespective of its topology. Models are on the X-axis; on the Y-axis, we report percentages for RR, U, and W.

We simulated 5-gene, heterogeneous, concatenated alignments of 7,740 sites (each gene being 1,548 sites), to test whether partitioned models can be used as an alternative to mixture models in analyses of heterogeneous datasets. These datasets were simulated in Phylobayes (BLMF = 1) using the Felsenstein tree only, see Methods for details. The partitioned models were found to behave like homogeneous models having a very poor RRs (RR_Partition_ = 38), worse than RR_MLHo_ = 49 and RR_GTR_ = 45. C10 (for these analyses specified as LG + F + G + C10 because the simulated Phylobayes datasets best fit LG-based models), MLHe (similarly specified as LG + F + G + CXX models) and CAT-GTR achieve much higher RRs (RR_LG-C10_ = 78, RR_MLHe_ = 90 and RR_CAT-GTR_ = 96), Fig. 3 and Table S3.

#### Heterogeneous models outperform homogeneous models

**Figure 4:**
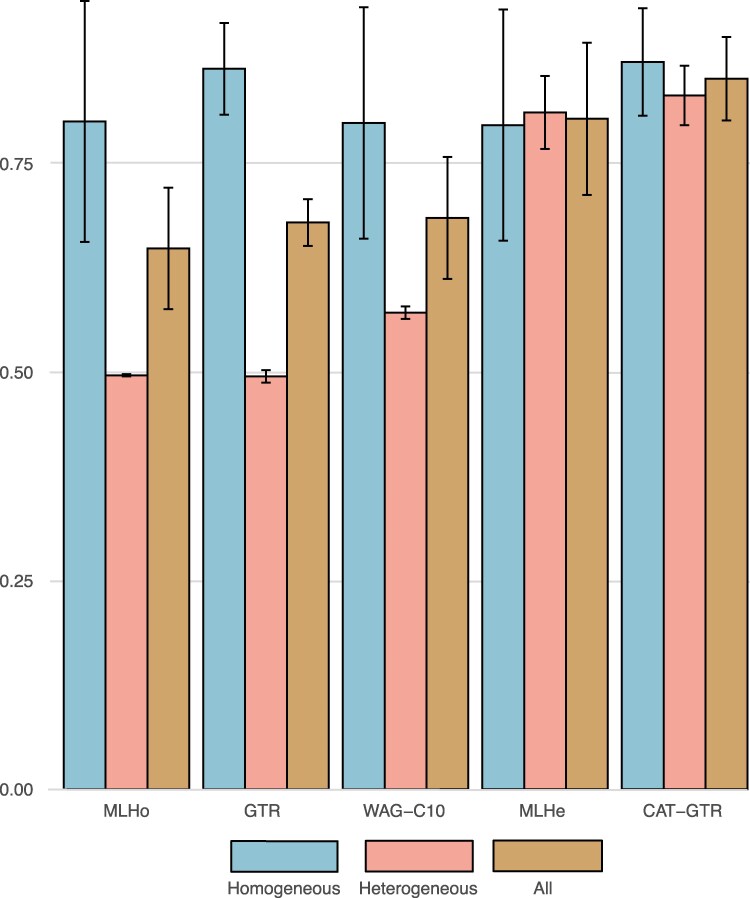
The performance of the considered models. Performance calculated over the Elynx simulated datasets. The values used in the calculations are those in Tables S1 and S2. Performance values are calculated averaging across all experimental conditions: dataset sizes, BLMFs, and target topologies. Light blue: homogeneous datasets only. Pink: heterogeneous datasets only. Brown: all datasets (heterogeneous plus homogeneous). Models are on the X-axis; on the Y-axis we report the performance score. Bars represent the proportion of unresolved phylogenies.

To summarize the results in Fig. 2 in a more intuitive way, we estimated what we call the “Performance” (P) of every model we tested using Elynx simulated datasets. Performance is calculated as the RR minus the error rate, the proportion of wrongly recovered trees. Accordingly, P = RR-W; Tables S1 and S2. We averaged these values across all experimental conditions (BLMFs, trees, and dataset size) for (1) both the homogeneous and heterogeneous datasets, (2) the homogeneous datasets only, and (3) the heterogeneous datasets only (reported in Fig. 4 and Table S4). These results show that homogeneous models perform well when used to analyze homogeneous data (with GTR doing better than MLHo) but perform poorly with heterogeneous data. With homogeneous data, the performance of WAG-C10 and MLHe is the same as that of MLHo. However, with heterogeneous data, WAG-C10 and MLHe perform better than MLHo and GTR. Furthermore, MLHe performs better than WAG-C10 with heterogeneous data. CAT-GTR performs better than all the other considered models, performing as well as GTR (and better than MLHo, MLHe, and WAG-C10) with the homogeneous data, and better than all the other models with the heterogeneous data.

#### Analysis of the branch lengths of the Farris tree

We tested the ability of alternative models to correctly infer the lengths of key branches on the Farris tree for the large, Elynx datasets. The key branches are those subtending the two long-branched clades, referred to as the (AB)-branch and (EF)-branch or just (AB) and (EF), and the short branch subtending the clade including A, B, E and F, referred to as the (ABEF)-branch or just (ABEF), see Fig. 1a. The goal of these analyses is to test whether different models infer the Farris tree, when it is the simulation target, because they correctly interpret the substitution history along its branches or because they confuse parallel substitutions along (AB) and (EF) for substitutions along (ABEF), thus overestimating the evidence, length, and support for (ABEF) as expected if these models are negatively affected by LBA, see Methods and Swofford et al. (2001) for details.

**Figure 5:**
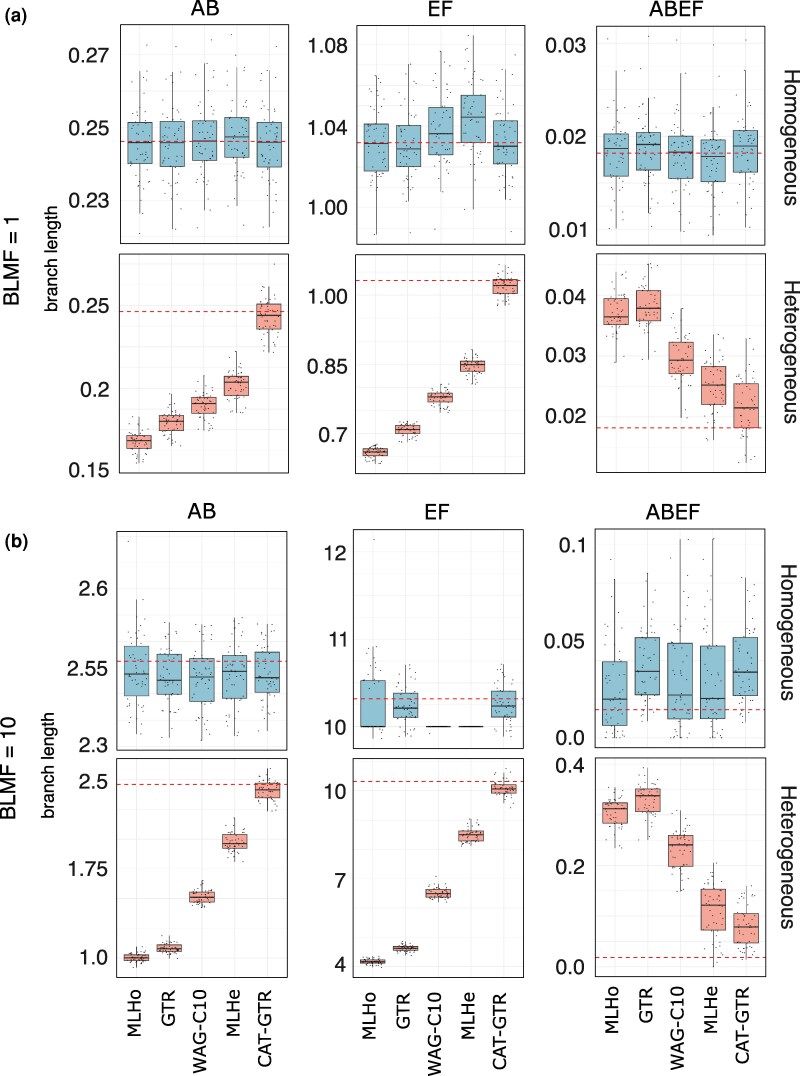
The lengths of the branches subtending the (AB), (EF), and (ABEF) clades in Farris trees. This test is performed for the large, homogeneous and heterogeneous datasets simulated on trees with BLMF = 1 and BLMF = 10 (Fig. 1a and Figure S5). a) Results obtained when BLMF = 1. b) Results obtained when BLMF = 10. Light blue: homogeneous datasets. Pink: heterogeneous datasets. The red dotted lines indicate the target branch lengths. Models are on the X-axis; on the Y-axis, we report branch lengths.

When BLMF = 1 and the data are homogeneous, all models correctly infer all target branch lengths, although WAG-C10 and MLHe tend to mildly overestimate the length of (EF)—the longest branch in the datasets, Fig. 5a and Table S5. With homogeneous data and very long branches (BLMF = 10), MLHo, WAG-C10, and MLHe strongly underestimate the median length of (EF), Fig. 5b. However, the distribution of values in the interquartile range of MLHo is broad and includes the target length, Fig. 5b and Table S5. Differently, the interquartile ranges of WAG-C10 and MLHe have no variance (Fig. 5b and Table S5). The (unrealistic) precision of the (EF) estimates under WAG-C10 and MLHe when BLMF = 10 leads these models to significantly underestimate (EF). GTR and CAT-GTR mildly overestimate the length of (ABEF), but the target lengths for all key branches fall within the range of the estimates inferred by these models, Fig. 5b and Table S5. For the heterogeneous datasets (Fig. 5a and b and Table S5), MLHo and GTR significantly underestimate the lengths of the long-branched (AB) and (EF) lineages and significantly overestimate the length of (ABEF): the target values never fall within the range of branch lengths inferred by these models for both BLMF = 1 and BLMF = 10. Heterogeneous models do progressively better (with both BLMF = 1 and BLMF = 10), with CAT-GTR providing good estimates for all key branch lengths, Fig. 5a and b and Table S5, even though at BLMF = 10 also CAT-GTR tends to mildly overestimate the length of (ABEF) and mildly underestimate (EF).

#### Model fit tests

We tested the ability of the models in our study to fit the across-site compositional heterogeneity of our datasets using model adequacy tests. For the models we implemented using Phylobayes (GTR and CAT-GTR), we used PPA. For the models implemented in IQTree2 (MLHe, WAG-C10, LG-C10, MLHo, and partitioned models) we used PB—as in Giacomelli et al. (2025). All Z-Score presented are averages. As shown above (results section: *Datasets simulated in our study*) GTR provided a good fit to the homogeneous datasets but failed to fit the heterogeneous ones. Similarly, MLHo fits our homogeneous small and large (Elynx) datasets (PB-Z-Score_MLHo_ = −0.64 – SD = 0.21 and PB-Z-Score_MLHo_ = −0.016 – SD = 0.28) but fails to fit the heterogeneous large and small (Elynx) datasets (PB-Z-Score_MLHo_ = 5.21 – SD = 0.55 and PB-Z-Score_MLHo_ = 10.12 – SD = 0.21). MLHo also fails to fit the 5-gene datasets (PB-Z-Score_MLHo_ = 17.34 – SD = 2.35). WAG-C10 and MLHe, similarly to MLHo and GTR, provided a good fit to our homogeneous datasets (PB-Z-Score_WAG-C10_ = −0.84 – SD = 0.15 and PB-Z-Score_MLHe_ = −1.11 – SD = 0.45, small datasets and PB-Z-Score_WAG-C10_ = 0.012 – SD = 0.44 and PB-Z-Score_MLHe_ = −0.22 – SD = 0.61, large datasets). WAG-C10 fits some of our small (Elynx) heterogeneous datasets (PB-Z-Score_WAG-C10_ = 2.26 – SD = 0.41) but fails to fit our large ones (PB-Z-Score_WAG-C10_ = 5.05 – SD = 0.43). MLHe fits relatively well the small (Elynx) datasets (PB-Z-Score_MLHe_ = 1.91 – SD = 0.4) but not the large ones (PB-Z-Score_MLHe_ = 3.92 – SD = 0.26). Partitioned models fail to fit the 5-gene datasets (PB-Z-Score_Partition_ = 19.64 – SD = 1.42). Similarly, LG-C10 and MLHe fit poorly these datasets (PB-Z-Score_LG-C10_ = 15.71 – SD = 1.86 and PB-Z-Score_MLHe_ = 13.91 – SD = 0.76).

CAT-GTR fits all our datasets well. With the Elynx small and large homogeneous datasets CAT-GTR has PPA-Z-Score_CAT-GTR_ = −0.006 (SD = 0.31) and PPA-Z-Score_CAT-GTR_ = 0.26 (SD = 0.11), respectively. With the heterogeneous Elynx small and large datasets and the 5-gene datasets, CAT-GTR has PPA-Z-Score_CAT-GTR_ = 0.32 (SD = 0.26), PPA-Z-Score_CAT-GTR_ = 0.15 (SD = 0.2) and PPA-Z-Score_CAT-GTR_ = 0.90 (SD = 0.22), respectively. See Allresults.xls (https://doi.org/10.6084/m9.figshare.29404982).

#### Evaluating the number of site-frequency categories used by CAT-GTR

**Figure 6:**
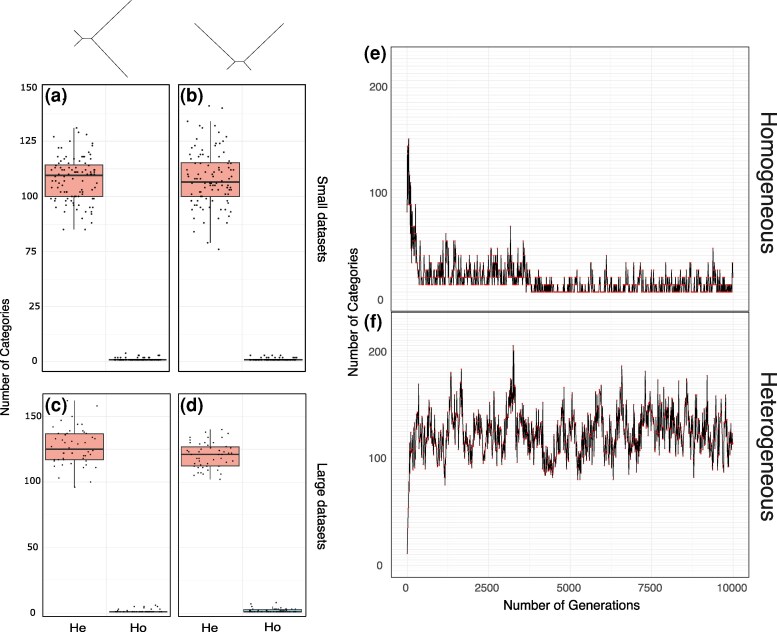
Number of categories used by CAT-GTR analyses. a) Elynx simulated small datasets (Farris tree; BLMF = 1; burnin = 5,000 cycles). b) Elynx simulated small datasets (Felsenstein tree; BLMF = 1; burnin = 5,000 cycles). c) Elynx simulated large datasets (Farris tree; BLMF = 1; burnin = 5,000 cycles). d) Elynx simulated large datasets (Felsenstein tree; BLMF = 1; burnin = 5,000 cycles). Light blue: homogeneous datasets. Pink: heterogeneous datasets. He = Heterogeneous data; Ho = Homogeneous data. e) Plot illustrating how the number of categories used by CAT-GTR analyses changes as the MCMC analysis progresses, for a randomly selected large homogeneous dataset simulated using the Farris tree (BLMF = 1). Note that after convergence the number of categories used is most frequently one, even if the parameter space is still explored, as expected in a MCMC chain. f) Plot illustrating how the number of categories used by CAT-GTR analyses changes as the MCMC analysis progresses, for a randomly selected large heterogeneous dataset simulated using the Farris tree (BLMF = 1). Note that in this case the number of site-frequency profiles used does not drop to one after convergence.

We investigated the number of site-frequency profiles used by CAT-GTR in the analyses of homogeneous and heterogeneous datasets (BLMF = 1). We calculated, from the trace files of the CAT-GTR analyses, the mode (*m*) of the number of site-frequency profiles used (*Nmode* parameter) after burnin (Fig. 6 and Table S6). For the small (Elynx) datasets when the datasets are homogeneous, *m_Nmode_* = 1 (range = 1 to 4; Fig. 6a and b). For the large datasets, *m_Nmode_* = 1 (range = 1 to 8; Fig. 6c and d). For both the large and small datasets, the average number of categories is slightly higher than 1 (Table S6—as implied by the range values). For the heterogeneous small (Elynx) datasets *m_Nmode_* = 87 (range = 51 to 119; Fig. 6a and b) and for the large heterogeneous datasets *m_Nmode_* = 122 (range = 96 to 162; Fig. 6c and d). The number of generations that randomly selected Bayesian CAT-GTR analyses (from one homogeneous and one heterogeneous dataset) took to achieve convergence, and the numbers of components these analyses use after convergence are shown in Fig. 6e and f. A comparison of Fig. 6e and f shows that for homogeneous data, after convergence, most generations use one site-frequency profile only. For heterogeneous datasets, the number of categories used never drops to one. Rather, it stabilizes within a limited range of values that is finite and significantly lower than the theoretical maximum (i.e. the number of sites in the alignment) for our large datasets. We do not report results in tables and figures for the 5-gene datasets. However, these datasets had *m_Nmode_* = 209 (Range = 184 to 262). See Allresults.xls (https://doi.org/10.6084/m9.figshare.29404982) for all our results and output files.

#### Investigating the weights of the WAG + F components in WAG + F + G + CXX analyses

When CXX-based models are set using the + F flag (e.g. WAG + F + G + CXX or LG + F + G + CXX—more generally referred here as “GTR”+F + G + CXX-like models), a component is set to represent “GTR + F” (in our case WAG + F). For all the WAG + F + G + C10 (WAG-C10) and MLHe (WAG +F + G + C60) analyses performed using homogeneous datasets, we extracted the components' weights (as optimized by IQTree2). For both WAG-C10 and MLHe, the average weight for the WAG + F component (across both tree topologies and all considered BLMF values) was ∼1. For WAG-C10 (small and large datasets respectively): Weight_WAG_  _+_  _F_ = 0.98 (SD = 0.005) and Weight_WAG_  _+_  _F_ = 0.99 (SD = 0.003). For MLHe, Weight_WAG_  _+_  _F_ = 0.97 (SD = 0.089) and Weight_WAG_  _+_  _F_ = 0.98 (SD = 0.005), respectively. All the other components had weights of approximately zero. This implies that virtually all sites in the alignments were modeled using (WAG + F + G), the + G part of the model being considered independently.

## Discussion

### The ability of models to recover the target tree depends on the heterogeneity of the data

To interpret results of simulations, it is important to understand whether the simulated datasets are realistic, which with reference to the focus of our study means that the heterogeneity of the simulated data should be comparable to that of real data. Information exists about the heterogeneity of published phylogenomic datasets covering different lineages and evolutionary depths, e.g. (Tarver et al. 2016; Feuda et al. 2017; Giacomelli et al. 2022, 2025; Cai et al. 2024; Tao et al. 2026), indicating that empirical phylogenomic datasets are usually heterogeneous. In agreement with these results, the empirical 32-gene alignment used to parametrize our simulations had PPA-Z-Score_GTR-realdata_ = 11.39, confirming its heterogeneity. Our small (Elynx) heterogeneous datasets had PPA-Z-Score_GTR_∼ 6 and the large ones PPA-Z-Score_GTR_∼ 12. The small (Phylobayes) 5-gene datasets had PPA-Z-Score_GTR_∼ 11. These values demonstrate that our simulated datasets are heterogeneous and should be informative about the behavior of the considered models with real-world, phylogenomic, datasets.

PPA-Z-Scores can only be compared for datasets of comparable size. Our 32-gene, model dataset and our small Elynx simulated datasets have the same size (7,743 sites and 10 taxa). Our Phylobayes simulated datasets are for all intents and purposes also of the same size (7,740 sites and 10 taxa). This allows us to meaningfully compare PPA-Z-Scores for these datasets, which tells us that (1) different software simulated data with different levels of heterogeneity, (2) Elynx simulated data are less heterogeneous than Phylobayes simulated data, and (3) Phylobayes simulated data have a level of heterogeneity directly comparable to that of the real dataset (PPA-Z-Score_GTR-realdata_ = 11.39 and PPA-Z-Score_GTR-PhylobayesSimdata_ = 10.86). That is, with reference to across-site compositional heterogeneity, Phylobayes simulated datasets are more realistic than Elynx simulated datasets. These results are interesting because they inform the interpretations of our simulations. All our analyses of small datasets simulated on trees with BLMF = 1 (Figs. 2b and 3—Phylobayes datasets were only simulated using BLMF = 1, see Methods), achieved lower RRs and higher error rates as heterogeneity increased, i.e. when used to analyze Phylobayes simulated data. However, the relative performance of different models, with datasets simulated with different software (and hence different level of heterogeneity), remains unchanged. We thus expect that the pattern of performance emerging from our analyses should be representative of the performance that we can expect, from the tested models, with real-world empirical datasets. Except that, as real datasets will be more difficult to analyze than simulated ones (Trost et al. 2024), error rates will be proportionately higher and RRs lower.

### The tested models variably fit our heterogeneous datasets

Model fit tests indicate that not all models fit our heterogeneous datasets equally well. Homogeneous models (GTR and MLHo) and the partitioned models fail to describe our heterogeneous datasets. C10-based models do better but still fail to fit most datasets, adequately describing only a subset of the small, Elynx datasets (PB-Z-Score_WAG-C10_ = 2.26 – SD = 0.41). MLHe does better than WAG-C10 but when the heterogeneity in the data increases (5-gene datasets), this strategy also fails (PB-Z-Score_MLHe_ = 13.91 – SD = 0.76). The only modeling strategy that adequately fits all our heterogeneous datasets is CAT-GTR.

CAT-GTR is a non-parametric modeling strategy, exploring models with different numbers of mixture components to fine-tune the model to the heterogeneity of the data (Fig. 6). Accordingly, the ability of CAT-GTR to fit all our heterogeneous datasets better than the other models we tested (CXX-based models included) is not surprising. However, CAT-GTR also adequately describes our homogeneous datasets, which might be seen as more surprising. Yet, this result agrees with theoretical expectations. GTR is nested within CAT-GTR, as it can be defined as a CAT-GTR model using one site-frequency profile. Figure 6e shows how the CAT-GTR analysis of a randomly selected homogeneous dataset explores the parameter space for *Nmode*, the number of site-frequency profiles used at each iteration of the MCMC chain. CAT-GTR starts by considering models with up to ∼150 components, which is much less than the theoretical maximum for our dataset. However, as the MCMC chain progresses fewer components are used. Convergence is reached after ∼2,500 generations, Fig. 6e. Very few site-frequency profiles are used after convergence, with most MCMC generations using only one site-frequency profile (Fig. 6e). As it is customary for Bayesian MCMC chains, the parameter space continues to be explored after convergence (hence the average and range of *Nmode* remain higher than one). Nonetheless, for all the chains we run on homogeneous datasets (Fig. 6a–d) *m_Nmode_* = 1. That is, after convergence CAT-GTR is using a single site-frequency profile on most MCMC cycles, converging to a homogeneous GTR model. These conclusions are reinforced by our PPA showing that GTR and CAT-GTR comparably fit our small (average Z-Score_GTR_ = 0.017 and average Z-Score_CAT-GTR_ = −0.006) and large (average Z-Score_GTR_ = 0.38 and average Z-Score_CAT-GTR_ = 0.26) homogeneous datasets. Finally, Fig. 4 clearly shows that GTR and CAT-GTR have the same performance with homogeneous data. We conclude that with homogeneous data CAT-GTR converges to an appropriate homogeneous model (GTR), efficiently avoiding overparametrization.

When analyzing homogeneous datasets using IQTree2, we set MLHe and WAG-C10 as WAG + F + G + CXX (with CXX = 10 or 60). When CXX models are set in this way (using + F), IQTree2 assigns WAG + F to one of the CXX components and estimates all components' weights from the data. An inspection of the IQTree2 output files shows that the weight assigned to the WAG + F component was (on average across all our datasets) larger than 0.97 out of 1 (for both WAG-C10 and MLHe). All the other components have weights very close to zero. WAG + F dominated the estimation of the likelihood, implying that, similarly to CAT-GTR, also WAG + F + G + C10 and WAG + F + G + C60 converged to an appropriate homogeneous model (WAG + F + G—the + G part of the model always being considered) when faced with homogeneous datasets. This is consistent with the results in Fig. 4, showing that with homogeneous datasets the performance of MLHo (which mostly selected WAG + G as the best fit model—as expected given how the data were simulated), MLHe and WAG-C10 are indistinguishable, and results of PB analyses showing that MLHo, MLHe, and WAG-C10 fit comparably well our homogeneous datasets: −1.11 < PB-Zscore < 0.012 across all three models and across all our homogeneous datasets. These results are in agreement with and confirm the conclusions of previous papers by Cai et al. (2024) and Banos et al. (2024a), suggesting CAT-based and CXX-based models should not be expected to overparametrize the data.

Li et al. (2021) suggested that CAT-based models use too many poorly populated components, thus overfitting the data, and that CXX-based models should be preferred instead. However, the rationale underpinning the argument of Li et al. (2021) is difficult to reconcile with the fact that components describing smaller proportions of sites contribute less to likelihood calculatons, which should shield both CAT-based models and CXX-based models from overparametrization (Banos et al. 2024a). Li et al. (2021) did not provide experimental evidence (e.g. simulations) to support the general validity of their conjecture, and there is no published evidence indicating that overparametrization is a major problem in CAT-based analyses and Bayesian phylogenetics more broadly (Lemmon and Moriarty 2004; Guimaraes Fabreti and Hohna 2023). The possibility that CAT-based models might overparametrize the data is not even mentioned in Whelan and Halanych (2017), despite this study suggesting that CAT-GTR does not perform better than homogeneous and partitioned models. Our results contradict predictions from the study of Li et al. (2021) study. If these authors were correct, with homogeneous data, CAT-GTR should be overparametrized and perform poorly. Yet, we show that the opposite is true. Both CAT-GTR and CXX-based models are effective at avoiding overparametrization, as expected based on the argument of Banos et al. (2024a). CXX models only use a limited number of categories (ranging from 10 to 60), differently from the non-parametric CAT-GTR. Our results show that this can limit the ability of CXX models to fit the data, and at least in our simulations, this correlates with a drop in accuracy for these models (compare Fig. 2b—results for BLMF = 1, and Fig. 3).

To conclude, we did not find any evidence that CAT-GTR overparametrizes the data. However, we were able to demonstrate that as the data become more heterogeneous CXX-models become underparametrized, differently from CAT-GTR. This might be a problem when CXX-based models are used to analyze real-world datasets, particularly those designed to resolve tricky nodes, which are usually (perhaps invariably) heterogeneous and generally include long-branched taxa.

It is important to note that to allow for a fair comparison of commonly used homogeneous and heterogeneous models, we only tested models with the same elements: one GTR matrix, one or more site-frequency profiles, and a Gamma distribution. Accordingly, CAT-Poisson and Poisson-CXX models were not tested. These models use a Poisson process rather than a GTR matrix to account for AA replacement heterogeneity, and this would have made their comparison with the other models in our study not obvious. For example, Poisson-based models should not be expected to converge to a homogeneous model when analyzing our homogeneous datasets. Our homogeneous datasets are AA replacement heterogeneous (having been simulated under WAG + G) and Poisson-based models will have to use a mixture of profiles to account for this form of heterogeneity. However, as Poisson-based models do not use GTR to account for AA replacement heterogeneity, their use of a mixture of components would have been appropriate, rather than a symptom of overparametrization. Poisson-based models use a different, simpler way to concomitantly account for all forms of site-heterogeneity in the data, and it is not possible to quantify how many components a Poisson-based model would need to adequately account for the AA replacement heterogeneity in our homogeneous datasets: different combinations (characterized by different weights) might be possible. Accordingly, generalizing about the behavior of Poisson-based models with homogeneous data would not have been possible. A full comparison of Poisson-based heterogeneous models against the heterogeneous models in our datasets would certainly be interesting but is outside the scope of this study.

### CAT-GTR outperforms homogeneous, partitioned and CXX models

Our results show that homogeneous models can be expected to perform well only when used to analyze homogeneous data (Figs. 2 and 4). With heterogeneous data, homogeneous models perform relatively well only when heterogeneity is low (Elynx datasets), and branch lengths are short (BLMF = 0.1 or 1; Fig. 2). When the branch lengths of the target trees are increased (BLMF = 5 or 10), the ability of homogeneous models to infer the target tree drops dramatically (Fig. 2). Similarly, when heterogeneity is increased to more realistic levels (the 5-gene datasets), the ability of homogeneous models to recover the correct tree worsens, despite LBA not being experimentally exacerbated in these datasets, compare results in Fig. 2 (Felsenstein tree and BLMF = 1) against those in Fig. 3.

Turning our attention to the mixture models, it is evident that, with heterogeneous data, they perform much better than homogeneous and partitioned models (Figs. 2–4). However, Fig. 4 (see also Figs. 2 and 3) indicates that not all mixture models perform equally well. At the least in our analyses, C10-based models are the worst performing ones and CAT-GTR is the best performing one.

It is interesting to note that not all heterogeneous models infer the branch lengths of the Farris tree correctly with homogeneous datasets. Figure 5 shows that when BLMF = 10, WAG-C10 and MLHe significantly underestimate the length of the longest branch in the Farris tree (EF). However, the similarity of the WAG-C10 and MLHe results (Fig. 5) is a consequence of the fact that these models converged to the same homogeneous model (WAG + F + G) in these analyses. Hence, it is not necessarily the use of heterogeneous models that led to these incorrect estimates. Indeed, an inspection of the results obtained for MLHo, which almost always identified WAG + G as best fit (see Allresults.xls https://doi.org/10.6084/m9.figshare.29404982) shows that also in the case of this model the median branch length of (EF) is severely underestimated, even though a diffuse distribution of values around the median is found with this model (Fig. 5). This might be interpreted to suggest that the use of WAG + G could be responsible for the underestimation of (EF), but not for the unrealistically small distribution of (EF) lengths around the median in MLHe and WAG-C10 analyses at BLMF = 10. We conclude that the use of + F in the MLHe and WAG-C10 analyses, which converged to WAG + F + G models rather than WAG + G models, could have led to these unrealistically narrow distributions of branch lengths, although we do not have an explanation for why this might be the case. Importantly, CAT-GTR also converges to a homogeneous model (GTR) with homogeneous datasets (see above), and we also run analyses directly set to use the homogeneous GTR model (Fig. 5). Neither of these analyses underestimated the length of (EF) with homogeneous data when BLMF = 10, see Fig. 5. Accordingly, irrespective of what exactly caused the underestimation of (EF) with MLHo, MLHe and WAG-C10, and the unrealistically narrow distribution of (EF) values with WAG-C10 and MLHe (BLMF = 10), it is evident that GTR is more effective than WAG-based models at estimating branch lengths with our homogeneous datasets. With heterogeneous datasets WAG-C10 and MLHe do not show the artifactual behavior they show with homogeneous data, Fig. 5. However, with these datasets CAT-GTR still performs best.

### The Felsenstein tree is harder to resolve and there is nothing to gain from using homogeneous models with heterogeneous data

Our results identify genuine differences in the observed patterns of discovery for the Farris and the Felsenstein trees. The Farris tree (Fig. 2a and c) seems harder to identify with homogeneous small datasets. However, with these datasets, the tree is unresolved rather than incorrect and the RR improves when more data are used. Inspection of the branch lengths inferred using homogeneous data for the Farris tree (Fig. 5) clearly shows that with homogeneous datasets the Farris tree is usually inferred for the correct reason, i.e. because the substitution history is correctly interpreted by the model (but see above the MLHo, WAG-C10 and MLHe cases when BLMF = 10). Irrespective of that, our results indicate that difficulties in recovering the Farris tree with small homogeneous datasets are not caused by systematic errors and adding more data usually solves the problem. With heterogeneous data, the Farris tree is very efficiently inferred by homogeneous models, but an inspection of tree lengths in Fig. 5 illustrates that with these datasets, the efficiency of the homogeneous models is driven by LBA. For the Felsenstein tree, the pattern is very different. This tree is harder to recover with heterogeneous datasets, and the RR decreases when using more data, indicating that the problem is systematic and adding data makes it worse. With heterogeneous data, the ability to recover the Felsenstein tree is clearly model dependent, with this tree becoming impossible to recover, with homogeneous and poorly fitting heterogeneous (e.g. WAG-C10) models, when the effect of LBA is experimentally exacerbated. It could be argued that our results suggest that the utility of different models is target-tree dependent, with homogeneous models being better suited to analyze data that evolved under the Farris tree. However, this would be a misinterpretation, as homogeneous (and underparametrized heterogeneous) models incorrectly estimate the branch lengths and overestimate the support for the Farris tree, even when it is the simulation target.

Swofford et al. (2001) investigated the case of the relative efficacy of parsimony and model-based likelihood inference at recovering the Farris tree. Swofford et al. (2001) study is analogous to ours and these authors achieved similar conclusions, with the difference that in their analyses it was parsimony that displayed the negative features we observe when homogeneous and underparametrized heterogeneous models are used to model heterogeneous data. Our results compare well with those of Swofford et al. (2001), and similarly to these authors we conclude that despite the apparent efficacy of homogeneous and underparametrized heterogeneous models at inferring the Farris tree, there is nothing to gain in using these models, not even when the Farris tree is target and LBA is not experimentally exacerbated (Fig. 5a) because these models are not *honest* (*sensu* Swofford et al. (2001)) and *pretend* (*sensu* Swofford et al. (2001)) that the Farris tree is well supported despite the majority of that support being the consequence of a bias.

CAT-GTR is more robust to LBA than the other considered models and correctly approximates the substitution history along the branches of the Farris tree also when LBA is experimentally exacerbated (Fig. 5b). Of the models we tested CAT-GTR is therefore the only one that seems to have the power to honestly assess the support for the Farris tree when it is the simulation target and the data are heterogeneous. CAT-GTR performs best also with the Felsenstein tree. Furthermore, we demonstrated that CAT-GTR performs well also with homogeneous data (Fig. 4), even though, given that CAT-GTR uses a GTR matrix, a mixture of site-frequency profiles is not necessary for CAT-GTR to model these datasets. We were able to show that this happens because with homogeneous datasets CAT-GTR avoids overparametrization instantiating a model using (for most MCMC iterations) one site-frequency profile only, thus converging to GTR. The ability of CAT-GTR to converge to GTR is a consequence of its non-parametric nature, which in our opinion is a clear advantage of this model, setting it aside from the other models tested in our study. However, it should be noted that recent “GTR” + F + G + CXX-like implementations of CXX models also allow convergence to an appropriate homogeneous model, when the data are homogeneous, by downweighing components.

Overall, our results clearly suggest that in all cases where the implementation of CAT-GTR is computationally feasible, this model should be preferred. However, we note that all considered models tend to underestimate the length of the long-branched taxa in the Farris tree while overestimating that of the short branch subtending them (Fig. 5). This indicates that all considered models (including CAT-GTR) are to a certain extent affected by LBA. CAT-GTR is simply more robust to LBA than the other tested models. This is consistent with our observation that, when BLMF = 10, also CAT-GTR finds it difficult to recover the Felsenstein tree (Fig. 2). Nonetheless, CAT-GTR is the only model in our study that might have the ability of inferring the true tree (branch lengths included) when a dataset is heterogeneous and affected by LBA, as it is usually the case for real-world datasets including tricky taxa.

It could be argued that in some of our experiments we exacerbated LBA to levels (BLMF = 10) that may not be observed in real data. However, the poor performance of homogeneous and partitioned models as well as the best performance of CAT-GTR is evident also with the Phylobayes simulated data, which are our most realistic datasets, both with reference to their heterogeneity and because in these datasets LBA had not been experimentally exacerbated (BLMF = 1).

### The results of Whelan and Halanych (2017) are uninformative

Whelan and Halanych (2017) agreed that CAT-GTR describes empirical substitution processes well. However, they continued that, this was irrelevant as their simulations showed that CAT-GTR did not generate trees that were more accurate than those that they inferred using Partitioned Models. Accordingly, they concluded that using CAT-GTR was unnecessary for accurate phylogenetic inference. Whelan and Halanych (2017) argument is regularly cited to dismiss the use of CAT-based models in phylogenomics, e.g. the recent study of Boudinot and Lieberman (2025). However, our results demonstrate that the conclusions of Whelan and Halanych (2017) are contingent on the properties of their simulated datasets and do not provide a reliable basis for informing real-world phylogenomic studies. Homogeneous and partitioned models are outperformed by all the mixture models we tested: CAT-GTR, MLHe and WAG-C10 (Fig. 3).

Whelan and Halanych (2017) did not test the heterogeneity of their datasets, assuming instead that their approach introduced heterogeneity, see Whelan and Halanych (2017) for details. We showed that 71% of the “heterogeneous” datasets of Whelan and Halanych (2017) are, to the contrary, homogeneous. This is a lower bound because some of their remaining datasets had Z-Score only slightly larger than 2 and could therefore be false positives.

Whelan and Halanych (2017) failed to provide a characterization of the performance of CAT-based models against heterogeneous datasets, and indeed their results are comparable to those we obtained with homogeneous datasets. When the data are homogeneous, which is certainly the case for at least 71% of Whelan and Halanych (2017) “heterogeneous” datasets, CAT-GTR converges to GTR (see above), and its performance becomes indistinguishable from that of GTR (as in Figs. 2–5), which is what Whelan and Halanych (2017) observed. Accordingly, their results do not constitute valid evidence to dismiss the use of CAT-based models.

## Conclusions

Real, concatenated, phylogenomic datasets seem to be invariably heterogeneous. Our simulations show that with these datasets, in the presence of LBA, the use of homogeneous and partitioned models leads to the recovery of artifactual phylogenies with high frequency. Accordingly, the use of these models to infer the main result of phylogenomic studies must be strongly discouraged.

CAT-GTR is the best performing of the models we tested, and the only one accurately inferring both target trees and their branch lengths at high frequency, with both homogeneous and heterogeneous datasets. Unsurprisingly, CAT-GTR is also the model with the best fit to all our simulated datasets, and we were able to confirm that it correctly converged to GTR (avoiding overparametrization) when the data are homogeneous. However, CAT-GTR can be computationally intractable for large datasets. While CXX-based models might replace CAT-based models in certain situations, their fit to the data should always be tested using PB (Giacomelli et al. 2025), as these models do not necessarily fit well every dataset and their accuracy, similarly to their fit, seems to decrease when heterogeneity increases. Other approaches such as CAT-PMSF (Szantho et al. 2023) have been developed to export parametric approximations of CAT-based models to ML. CAT-PMSF has been shown to have the potential to fit real datasets better than CXX-based models (Giacomelli et al. 2025; Tao et al. 2026) and might be a promising alternative to the implementation of CAT-based models when convergence is a problem. However, this approach is relatively new and needs further testing (Giacomelli et al. 2025). In addition, a diversity of other mixture models, not necessarily accounting for across-site compositional heterogeneity, have recently been developed (e.g., Crotty et al. 2019; Wong et al. 2024; Banos et al. 2024b; McCarthy et al. 2025) and their investigation should be strongly encouraged. GTRPmix (Banos et al. 2024b) seems a particularly promising alternative to standard CXX-models implementations and more extensive comparisons between CXX-based models, CAT-based models, GTRPmix, and CAT-PMSF are urgently needed.

Due to progress in genome sequencing we are entering a golden age of phylogenomics (Spang and Pisani 2026). This is both an exciting and challenging time, but only by embracing the modeling revolution that followed the introduction of mixture models (Lartillot and Philippe 2004), we will be able to truly harness the power of genomic data and improve our understanding of the tree of life.

## Methods

### The target trees

We used two (ten-taxon) target trees (Fig. 1), representing a topology in the Farris zone (Fig. 1a) and one in the Felsenstein zone (Fig. 1b). These trees are referred to in text as the Farris and Felsenstein trees. The branch lengths of these trees were first estimated using a real dataset (see below). We then modified the length of two lineages represented in light blue in Fig. 1 (the longest branches in the tree) to experimentally modulate the effect of long branch attraction (LBA; Figures S1–S6). The lengths of the blue branches were multiplied by 0.1, 1, 5 and 10. In text we refer to these multipliers as: branch length multiplier factors (BLMF). We note that multiplying branch lengths by one (BLMF = 1) does not change the lengths of the branches, which were originally estimated from the data (the trees in Fig. 1). However, by considering the trees in Fig. 1 as having been generated using BLMF = 1 helps to rationalize the description of our results in our figures and tables, where results are grouped by BLMF values. Accordingly, we present results obtained using four different pairs of trees characterized by having been generated using different multipliers: BLMF = (0.1, 1, 5, 10). BLMF = 0.1 shortens the light blue branches in Fig. 1 of one order of magnitude (Figures S1 and S2). The light blue branches in the BLMF = 5 trees (Figures S3 and S4) are five times longer than those of Fig. 1 but 50 times longer than those on Figures S1 and S2. The light blue branches in the BLMF = 10 trees (Figures S5 and S6) are one order of magnitude longer than those in Fig. 1, two orders of magnitude longer than those in Figures S1 and S2, but only twice as long as those in Figures S3 and S4.

### The Felsenstein vs. Farris tree system is informative about the robustness of models to LBA

The system formed by the Felsenstein and Farris trees in Fig. 1 and Figures S1–S6 represents a fundamental problem in phylogenetics which has been historically used to benchmark methods and models, e.g. (Sullivan and Swofford 2001; Swofford et al. 2001; Kapli and Telford 2020; Giacomelli et al. 2022). In this system the probability of inferring the Farris tree (Fig. 1a) increases as the branch lengths of the two light blue lineages (AB) and (EF) in Fig. 1 and Figures S1–S6 increase, even when the data were generated under the Felsenstein tree. This is the LBA problem, and it leads to different outcomes depending on whether the simulation target (i.e. the true tree) is the Felsenstein or the Farris tree. As the length of the two long-branched lineages (AB) and (EF) increases, parallel substitutions along these branches become more likely. Poorly fitting models mistakenly interpret these parallel substitutions as single substitutions representing evidence for the monophyly of (ABEF) instead (Swofford et al. 2001), irrespective of whether this node exists in the true tree (the Farris case) or not (the Felsenstein case). Accordingly, LBA manifests differently depending on whether the true tree includes the (ABEF) clade or not.

The Farris tree (Fig. 1a) includes the (ABEF) node. When this tree is the simulation target LBA manifests itself in the form of a correctly resolved Farris tree with incorrectly estimated branch lengths. This is because parallel changes along (AB) and (EF) are erroneously attributed to the branch subtending (ABEF), increasing its length, support and discoverability. This causes poorly fitting models to seem very efficient at inferring the Farris tree. However, the reassignment of substitutions from (AB) and (EF) to (ABEF) causes a systematic distortion of branch lengths. When the Farris tree is true, in the presence of LBA, the lengths of (AB) and (EF) are underestimated and that of (ABEF) is overestimated. Models that are robust to LBA correctly infer both the topology and the branch lengths of the Farris tree. The Felsenstein tree (Fig. 1b) does not include the (ABEF) node, but it still includes the long branches (AB) and (EF). As in the case of the Farris tree, LBA will cause parallel substitutions along (AB) and (EF) to be interpreted as evidence supporting a now non-existent (ABEF)-branch, leading to the erroneous recovery of the Farris tree of Fig. 1a, despite the Felsenstein tree of Fig. 1b being true.

Many studies tested the ability of models to recover the Felsenstein tree. Much less attention has been devoted to the Farris tree, perhaps because when the Farris tree is the target, we cannot distinguish between poorly performing and well-performing models based only on the topology of the inferred tree. Instead, we need to distinguish between models that infer the Farris tree for the correct reason (i.e. because they correctly interpreted the substitution history along the branches of the tree) from models that infer it for the wrong reason, i.e. because they incorrectly interpreted the substitution history (Swofford et al. 2001), and how to do that is less obvious.

Here we test homogeneous and heterogeneous models for their ability to recover both trees. To distinguish between models that infer the Farris tree for the correct reason (i.e. because they correctly infer the substitution history along the branches of the tree) from those that infer it for the wrong, or inappropriate *sensu* Swofford et al. (2001), reason (because they infer artifactual substitutions subtending (ABEF)), we distinguished between models that correctly infer the branch lengths of (AB), (EF), and (ABEF) from models that fail to do so (details below).

### Simulating single-partition homogeneous and heterogeneous datasets

We simulated homogeneous and heterogeneous datasets on every one of the target topologies in Fig. 1 and Figures S1–S6. To simulate the data, we needed a training dataset. This dataset was used to infer an initial set of realistic branch lengths for our two target trees (those in Fig. 1) and to estimate site-frequency profiles (one for each tree in Fig. 1) that we subsequently used to simulate heterogeneous datasets. We used a concatenation of 32 genes for 10 taxa subsampled from the D20 superalignment of Whelan et al. (2015). The 32 genes are listed in Allresults.xls (https://doi.org/10.6084/m9.figshare.29404982). The reason we only used 32 of the genes in D20 is because we did not want to include protein families where one or more of the 10 taxa was missing, as testing the effect of missing data on phylogenetic accuracy is outside the scope of our study. The ten subsampled taxa are as follows: 2 outgroups (*Capsaspora owczarzaki* and *Salpingoeca rosetta*), 2 sponges (*Oscarella carmela* and *Amphimedon queenslandica*), 2 ctenophores (*Mnemiopsis leidyi* and *Pleurobrachia bachei*), 2 cnidarians (*Aurelia aurita* and *Nematostella vectensis*), and 2 bilaterians (*Homo sapiens* and *Capitella teleta*). Whelan et al. (2015) dataset D20 was selected because there is independent confirmation that it is a good quality dataset (Feuda et al. 2017), and because it allowed selecting a set of taxa that can be naturally arranged into Farris and Felsenstein trees, given that the taxa it includes have heterogeneous branch lengths (Fig. 1). We note that while Whelan et al. (2015) dataset was assembled to study the metazoan phylogeny, this is irrelevant to our study, as we are only using it to parametrize the substitution process used in our simulations. We define the true tree that each simulated alignment supports (in terms of both topology and branch lengths) by fixing the true tree topology underpinning different sets of simulated datasets to be those reported in Fig. 1 and Figures S1–S6. To reinforce the point that the biological reality of the taxa in D20 is irrelevant, we replaced their names with letters (A to J) in our text and figures. Letter assignments followed the order in which the taxa are introduced above: A = *Capsaspora owczarzaki, … J*  *=*  *Capitella teleta.* We also note that we are aware that other more recent datasets are available for the same taxa, but for the scope of our study, this is irrelevant as we only need to extract from this 32-gene superalignment parameters to simulate realistic phylogenomic datasets.

The 32 genes were aligned using MAFFT—LINSI (Katoh and Standley 2013) and concatenated into an artificial “supergene” 7,743 sites long. This supergene was used to parameterize the models used in our simulations. We used Phylobayes MPI v.1.8 (Lartillot et al. 2013) to estimate the branch lengths of the Farris and Felsenstein trees in Fig. 1 using a WAG-CAT + G model. For both trees we ran constrained analyses. We ran two chains until they reached convergence, which was tested using *tracecomp*. For both topologies we summarized the trees sampled by the two chains using *bpcomp* (33,000 cycles; burnin = 10,000 cycles; subsampling frequency = 230). The branch lengths of the trees in Fig. 1 are from the consensus trees generated in Phylobayes using *bpcomp*. Both trees were fixed in analyses used to estimate site frequency profiles, in Phylobayes, using *read_pb* with the *-ss* flag (subsampling every 230 points). We used Phylobayes MPI v.1.8 to estimate the branch lenghts of the tree in Fig. 1, and both Phylobayes MPI v.1.8 and 1.9 to run the analyses. This is because the option to estimate the branch lenghts of fixed tree was removed from Phylobayes MPI v.1.9.

The eight trees in Fig. 1a and b, Figures S1–S6 were used to simulate across-site compositionally homogeneous and heterogeneous datasets of 7,743 sites (Small datasets—the length of the original concatenation of genes). In addition, we also generated larger, 30,942 site alignments (Large datasets) to evaluate how alignment-length influenced phylogenetic accuracy. Overall, we generated 32 sets of simulated datasets. Each set of small alignments included 100 datasets (for a total of 1,600 simulated datasets). Each set of large alignments included only 50 simulated datasets (for a total of 800 datasets), to reduce compute time and emissions. All small and large datasets were simulated using Elynx (Szantho et al. 2023). All simulations assumed the data to be AA replacement rate heterogeneous, and across-site rate heterogeneous (rates were sampled from a discrete Gamma distribution—alpha = 0.6 and number of rate categories = 4). For both homogeneous and heterogeneous datasets, AA replacement rate heterogeneity was introduced simulating the data using WAG. That is, the homogeneous datasets were simulated under a WAG + G model, and the heterogeneous datasets under a WAG-CAT + G model. We note that the choice of Elynx to simulate these datasets was dictated by the fact that this software allowed us to modulate the branch lengths of the long-branched taxa, which is something that cannot be done in Phylobayes. All simulated datasets, the site profiles inferred in Phylobayes and the scripts used to simulate the data are available: https://doi.org/10.6084/m9.figshare.29404982.

### Simulating partitioned datasets

We simulated a limited number of multigene datasets. To do so the 32-gene superalignment was used with the Felsenstein tree to run topologically constrained phylogenetic analyses in Phylobayes MPI v.1.8 under WAG-CAT + G, JTT-CAT + G, mtZOA-CAT + G, LG-CAT + G, and mtART-CAT + G. This was done to simulate 5 heterogeneous genes using different AA-replacement matrices. We used posterior predictive resampling to simulate (for each model) 100 datasets (each 7,743 sites long). All the alignments were then subsampled (using the jackknife) to include only 1,548 sites. The shorter (1,548 site-long genes) were then concatenated to generate 100, 7,740 sites (i.e. Small), superalignments of five genes each. These simulations were performed in Phylobayes (posterior predictive resampling) because Elynx can only simulate data using WAG. For the posterior predictive resampling, we used a burnin = 10,000. The Resampling frequency was calculated (for each model) to simulate 100 datasets from the chains after convergence. The 5-gene concatenated datasets are available (https://doi.org/10.6084/m9.figshare.29404982). These datasets were explicitly simulated to compare Partitioned Models against homogeneous and heterogeneous models. For these simulations we only used the Felsenstein tree, BLMF = 1 (to minimize computational time and emissions). This should be sufficient to allow testing whether partitioned models behave like homogeneous or heterogeneous models, given that the behaviour of all the other models is well defined by the analyses performed across the full diversity of our simulations.

### Estimating the ability of the tested models to account for compositional heterogeneity

We tested the ability of the models to describe the across-site compositional heterogeneity in our simulated datasets using model adequacy tests of amino acid diversity, e.g. Giacomelli et al. (2025). The fit of the models that we implemented in a Bayesian framework (GTR + G and CAT-GTR + G) was tested using posterior predictive analysis (PPA) in Phylobayes MPI v.1.9 (subsampling 100 points after convergence, burnin = 5,000 cycles, subsampling frequency = 50). For the other models: MLHo, Partitioned models, WAG-C10, and MLHe, that were implemented using maximum likelihood, model adequacy testing was performed in a maximum likelihood framework using the parametric bootstrap (PB). PB analyses used 100 simulated datasets (100 data points) and followed Giacomelli et al. (2025). Scripts and outputs for all these analyses are available at https://doi.org/10.6084/m9.figshare.29404982.

PPA analyses performed to test the fit of GTR + G were run, for the small datasets, on all simulated datasets. All other model adequacy tests (both PPA and PB) were run on a subsample of 5 small and large Elynx simulated homogeneous and heterogeneous datasets (20 datasets in total)—Felsenstein tree (BLMF = 1), and on 5 concatenated 5-gene datasets. As in Giacomelli et al. (2025) we consider that a model fits the data when −2 < Z-Score < 2.

### Phylogenetic analyses

For all datasets we performed analyses using CAT-GTR + G and GTR + G in Phylobayes MPI v1.9. We analyzed each dataset under their best fitting across-site compositionally homogeneous model (referred to in text and below as: MLHo—best fitting maximum likelihood homogeneous model) as estimated in IQTree2 (Minh et al. 2020) using the Bayesian Information Criterion (BIC) (Kalyaanamoorthy et al. 2017). All heterogeneous datasets were also analyzed under the best fitting model among: WAG + F + G and the following mixture models with fixed numbers of site-frequency categories (WAG + F + G + C10; WAG + F + G + C20; WAG + F + G + C30; WAG + F + G + C40; WAG + F + G + C50; WAG + F + G + C60), as estimated in IQTree2 using the BIC (referred to in text and below as MLHe—Best Fitting Maximum Likelihood Heterogeneous model). We note that with homogeneous datasets, BIC will never identify a mixture model as best fit. Accordingly, MLHe analyses for these datasets used a fixed WAG + F + G + C60 model. This allowed us to test the performance of CXX-based models with homogeneous datasets, which is important to test whether CXX-based models might overparametrize the data when alignments are not affected by compositional heterogeneity. When in IQTree2 models are set using the WAG + F + G + CXX form, the weights of the components are estimated from the data, and one component is assigned to WAG+ F. Hence, by setting the model to C60 we can test whether the heterogeneous model converges to a homogeneous WAG + F + G model downweighing all components but the WAG + F one, allowing testing whether MLHe can avoid overparametrization with homogeneous datasets. All datasets were also analyzed using WAG + F + G + C10, to evaluate the effect of using a mixture model with a very small number of fixed components. As in the case of MLHe, this model should be expected to converge to a WAG + F + G model when used to analyze homogeneous datasets. We note that for some heterogeneous datasets the MLHe model could be WAG + F + G + C10. Still, it is important to compare the effect of forcing the use of WAG + F + G + C10 on all datasets and compare the results against those inferred using MLHe, where the number of components in the model used will vary from one dataset to the next.

In addition to the analyses described above (MLHo, GTR + G; WAG-C10 + F + G; MLHe; CAT-GTR + G), the simulated 5-gene superalignments were also analyzed using Partitioned Models (in IQTree2). In these analyses each gene was modeled under its best fitting, across-site compositionally homogeneous model. Accordingly, for each analysis a partition file was set so that the 1,548 sites simulated under mtZOA-CAT + G were modeled using mtZOA + G, the 1,548 sites simulated under JTT-CAT + G using JTT + G etc. Each partitioned model used: WAG + G, JTT + G, mtZOA + G, LG + G, and mtART + G. For all datasets we estimated support using either ultrafast bootstrap (IQTree2–1,000 replicates), or Posterior Probabilities (Phylobayes).

For all Phylobayes analyses of our simulated datasets we ran one chain only, for 10,000 cycles, always using a burnin of 5,000 cycles. To make sure that the length of our chains was sufficient to achieve convergence, we tested the convergence achieved using these parameters for 10 of our small datasets and for 10 of our large datasets. In both cases, we used datasets simulated under the Felsenstein tree with BLMF = 1. For these 20 datasets, we ran two chains for 10,000 points and calculated convergence statistics using *bpcomp* and *tracecomp* (burnin = 5,000). These tests were run under CAT-GTR + G which always needs more cycles than GTR + G to achieve convergence.

As in Giacomelli et al. (2022) we defined the target tree to be correctly recovered when it was identified with support > 50%. When an alternative tree was recovered with support > 50% the analysis was deemed to have failed to infer the target tree. Analyses that achieved support values for the target tree (or for an alternative tree) < 50% were deemed indecisive (unresolved). We also estimated what we call the “Performance” of our models to intuitively summarize our results. This is the average, across all Elynx simulated datasets and all experimental conditions, of the difference between the “Recovery Rate” (the number of times a Farris or a Felsenstein tree was correctly recovered) and the “Error” rate (the number of times an analysis failed). We show the proportion of uncertain trees (support less than 50%) as a bar on top of the Performance in a histogram representation.

With reference to support, posterior probabilities might be higher than bootstrap values for the same dataset. We acknowledge that this might introduce a bias favoring CAT-GTR + G and GTR + G. While this is a possible limitation of our approach, we could not proceed in any different manner because CAT-GTR + G is only implemented in Bayesian software. Given that PP can be higher than bootstrap values and given that we wanted to compare the performance of CAT-GTR + G and GTR + G, particularly for homogeneous datasets, as CAT-GTR + G is expected to converge to GTR + G when the data are homogeneous, we also implemented GTR + G using Bayesian software. In this way, results from GTR + G and CAT-GTR + G analyses can always be readily compared. However, as CXX-based models are usually implemented using IQTree2, we preferred not to implement these models in a Bayesian framework. Similarly, as we want to compare MLHo with MLHe and WAG-C10 + G in the case of homogeneous datasets (to investigate whether also MLHe and WAG-C10 converge to an appropriate homogeneous model with homogeneous data), we implemented all these models in a ML framework using the same software. However, it does not seem that the use of posterior probabilities or ultrafast bootstrap proportions had a biasing effect on our results, as GTR + G and CAT-GTR + G are not always the models achieving greater support and our results are broadly comparable across models, irrespective of implementation.

In our analyses, CXX and CAT-based models are always combined with a Gamma distribution, and in the results, discussion and conclusion sections we will usually refer to the models we used as GTR, CAT-GTR, WAG and so on, without explicitly referring to the use of “+G” which is always implied. Only in a few cases, when it helps clarity, the “+G” part of the model is explicitly noted.

### Estimation of branch lengths on the Farris tree

We tested the ability of alternative models to correctly infer the lengths of key branches in the Farris tree for the Elynx simulated large datasets. The considered key branches are those subtending the two long-branched clades, the (AB)-branch and (EF)-branch, and the short branch subtending (ABEF)—the last common ancestor of (AB) and (EF) in Fig. 1a. This test was performed using Farris trees with BLMF = 1 (the tree in Fig. 1a) and BLMF = 10 (the tree in Figure S5), using both the homogeneous and heterogeneous datasets. The goal of this analysis is to understand whether, when our tested models infer the Farris tree, they infer it for the correct or the wrong reason. In brief, if a model infers the Farris tree because of LBA, the long-branched taxa—(AB) and (EF) will be underestimated, while the short branch underpinning (ABEF) will be overestimated, providing a way to discriminate between models negatively affected by LBA from models that infer the Farris tree (when it is target) because they are robust to LBA. See the Methods section “*The Felsenstein vs. Farris tree system is informative about the robustness of models to LBA”,* for details.

We used a Python script (available here: https://doi.org/10.6084/m9.figshare.29404982) to extract from our Farris trees the length of the (AB), (EF), and (ABEF). We plotted these values using boxplots and tested whether, for each considered model, the target branch lengths (reported in Fig. 1a and Figure S5), fell within the distribution of inferred values.

### Estimating the number of categories used by CAT-GTR

We extracted the number of site-frequency categories used at every cycle (post burnin) in our CAT-GTR analyses, to test whether CAT-GTR converges to a GTR model (i.e. a CAT-GTR model with one site-frequency category) when the data are homogeneous, e.g. Cai et al. (2024). The number of site-frequency categories used at each cycle of the MCMC chain in CAT-GTR analyses is stored in the trace files generated by Phylobayes (*Nmode* parameter). For this test we used the analyses performed for the datasets simulated using trees with BLMF = 1 (both Felsenstein and Farris trees). In detail, we used the following sets of simulated datasets: small and large homogeneous and heterogeneous datasets simulated on the trees in Fig. 1 using Elynx and the 5-gene superalignments generated using Phylobayes. For all the CAT-GTR analyses performed for these datasets we estimated the mode (*m*) of the site-frequency categories used after removing 5,000 data points representing the burnin. The mode is a better descriptor for the number of site-frequency profiles used after burnin than the average or the median, because MCMC chains are expected to keep exploring the use of different numbers of categories also after convergence. Accordingly, even if a CAT-GTR analysis converged to GTR, the mean and median number of components used would still be slightly higher than one. On the other hand, the mode of the distribution is expected to be equal to one, as most steps in the MCMC chains are expected to use one site-frequency category if the model correctly converged to GTR. For each experimental condition tested we had 100 (small datasets) or 50 (large datasets) data points. From these points we calculated averages, modes, and ranges.

### Estimating the weights of components used by WAG-C10 and MLHe analyses

To test whether WAG-C10 and MLHe analyses (set as WAG + F + G + CXX) converged to a homogeneous model when using homogeneous datasets, we extracted the weights estimated for the components of the model used in individual analyses and the weight of the WAG + F component. Expectations for this experiment are that if WAG-C10 and MLHe correctly converged to a homogeneous model (WAG + F + G), the weight assigned to the WAG + F component should be ∼1, with all the other components in the model having weights ∼ 0.

### Estimating the heterogeneity of Whelan and Halanych (2017) datasets

For all the heterogeneous datasets used by Whelan and Halanych (2017), we performed a Posterior Predictive analysis under GTR to evaluate whether this compositionally homogeneous model adequately fits them. For these datasets we ran full Bayesian analyses (2 chains until they reached convergence) in Phylobayes. The number of cycles and burnin changed from one dataset to the next—as these datasets have different numbers of sites and taxa and achieved convergence after different numbers of cycles. After that, for each dataset a PPA of across-site AA diversity (see above) was performed. For each dataset the subsampling frequency after convergence was independently estimated to sample 100 points for each dataset. We performed tests (see main text) also for a few key homogeneous datasets simulated by Whelan and Halanych (2017) to compare them against the results obtained for their heterogeneous datasets, and better understand whether their approach was successful at simulating heterogeneous datasets.

## Supplementary Material

msag090_Supplementary_Data

## Data Availability

All data and scripts can be found at https://doi.org/10.6084/m9.figshare.29404982.
