# Supplementary material for: Phylogenomic mixture models outperform homogeneous and partitioned models
Source: Mol Biol Evol. 2026 Apr 9;43(5):msag090. doi: 10.1093/molbev/msag090 (PMC13197666; doi:10.1093/molbev/msag090)

Fig. S6

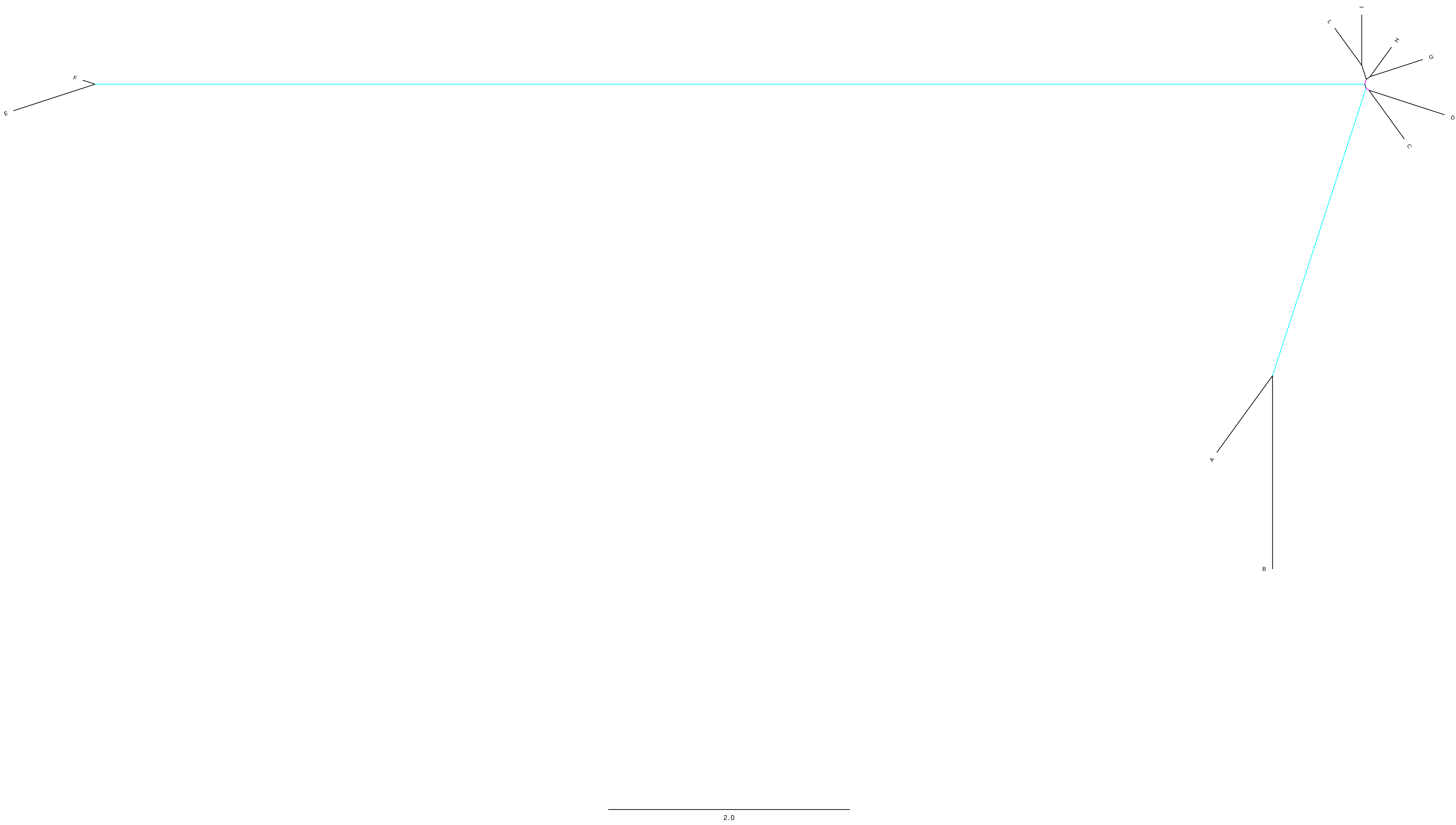

Supplement: msag090_Supplementary_Data [file msag090_supplementary_data.zip › FIGS6.pdf]
