# Supplementary material for: Phylogenomic mixture models outperform homogeneous and partitioned models
Source: Mol Biol Evol. 2026 Apr 9;43(5):msag090. doi: 10.1093/molbev/msag090 (PMC13197666; doi:10.1093/molbev/msag090)
Supplement: msag090_Supplementary_Data [file msag090_supplementary_data.zip › SM_mixture_sims_04-01-2026.pdf]

Supplementary information for

Phylogenomic mixture models accounting for across-site compositional heterogeneity outperform homogeneous and partitioned models

Davide Pisani, Mattia Giacomelli, Gergely J. Szöllősi, Maria Eleonora Rossi, Marc Domènech, Jesus Lozano-Fernandez

Fig. S1 Farris target tree (BLMF= 0.1). Light blue: long-branched lineages. In Pink short-branched lineages. For the target branch lengths see the Newick tree files in https://figshare.com/s/70fc81480557472bd970. Compare with Fig. 1a to see the effect on branch lengths of using BLMF= 0.1.

Fig. S2 Felsenstein target tree (BLMF= 0.1). Light blue: long-branched lineages. In Pink short-branched lineages. For the target branch lengths see the Newick tree files in https://figshare.com/s/70fc81480557472bd970. Compare with Fig. 1b to see the effect on branch lengths of using BLMF= 0.1.

Fig. S3 Farris target tree (BLMF= 5). Light blue: long-branched lineages. In Pink short-branched lineages. For the target branch lengths see the Newick tree files in https://figshare.com/s/70fc81480557472bd970. Compare with Fig. 1a to see the effect on branch lengths of using BLMF= 5.

Fig. S4 Felsenstein target tree (BLMF= 5). Light blue: long-branched lineages. In Pink short-branched lineages. For the target branch lengths see the Newick tree files in https://figshare.com/s/70fc81480557472bd970. Compare with Fig. 1b to see the effect on branch lengths of using BLMF= 5.

Fig. S5 Farris target tree (BLMF= 10). Light blue: long-branched lineages. In Pink short-branched lineages. For the target branch lengths see the Newick tree files in

https://figshare.com/s/70fc81480557472bd970. Compare with Fig. 1a to see the effect on branch lengths of using BLMF= 10.

Fig. S6 Felsenstein target tree (BLMF= 10). Light blue: long-branched lineages. In Pink short-branched lineages. For the target branch lengths see the Newick tree files in https://figshare.com/s/70fc81480557472bd970. Compare with Fig. 1b to see the effect on branch lengths of using BLMF= 10.

Table S1: The accuracy of different models with the small datasets

		Homo data						Hetero data					
	Tree	Felsenstein			Farris			Felsenstein			Farris		
BLMF	Model	RR	U	W	RR	U	W	RR	U	W	RR	U	W
	MLH	100	0	0	100	0	0	100	0	0	98	2	0
	GTR	100	0	0	100	0	0	100	0	0	100	0	0
	WAG-C10	100	0	0	100	0	0	100	0	0	99	1	0
	MLHe	100	0	0	100	0	0	100	0	0	100	0	0
0.1	CAT-GTR	100	0	0	100	0	0	100	0	0	100	0	0
	MLH	100	0	0	93	4	3	96	0	4	100	0	0
1	GTR	100	0	0	98	2	0	97	0	3	98	2	0

	WAG-C10	100	0	0	91	7	2	99	0	1	100	0	0
	MLHe	100	0	0	91	6	3	99	0	1	98	1	1
	CAT-GTR	100	0	0	98	2	0	99	1	0	97	1	2
	MLH	91	7	2	37	53	10	2	0	98	100	0	0
	GTR	88	3	9	75	20	5	0	0	100	100	0	0
	WAG-C10	93	5	2	35	56	9	29	1	70	99	1	0
	MLHe	95	4	1	30	60	10	66	7	27	76	19	5
5	CAT-GTR	89	4	7	75	20	5	66	9	25	92	7	1
	MLH	74	20	6	26	63	11	0	0	100	100	0	0
	GTR	60	14	26	62	33	5	0	0	100	100	0	0
	WAG-C10	77	14	9	32	58	10	7	8	85	98	1	1
	MLHe	75	17	8	33	55	12	50	11	39	67	24	9
10	CAT-GTR	57	16	27	63	32	5	40	14	46	79	13	8

Table Legend: The table summarizes the Recovery Rates (the frequency with which the target (i.e. true) tree is recovered and the frequency with which unresolved (U) and incorrect (Wrong; W) trees are recovered, for the small datasets. BLMF is the Branch Lengths Multiplier Factor (see Methods, Main text and Fig. 1). Results are reported for all experimental conditions tested. We consider a tree to be unresolved when support (either posterior probability of ultrafast bootstrap) is lower than 50%, irrespective of whether the topology of the recovered tree is correct or not (see Fig. 2). All the data summarized in this table can be found in Allresults.xls (https://figshare.com/s/70fc81480557472bd970).

Table S2: The accuracy of different models with the large datasets

		Homogeneous data						Heterogeneous data					
	Tree	Felsenstein			Farris			Felsenstein			Farris		
BLMF	Model	RR	U	W	RR	U	W	RR	U	W	RR	U	W
	MLH	100	0	0	100	0	0	100	0	0	100	0	0
	GTR	100	0	0	100	0	0	100	0	0	100	0	0
	WAG-C10	100	0	0	100	0	0	100	0	0	100	0	0
	MLHe	100	0	0	100	0	0	100	0	0	100	0	0
0.1	CAT-GTR	100	0	0	100	0	0	100	0	0	100	0	0
	MLH	100	0	0	100	0	0	100	0	0	100	0	0
	GTR	100	0	0	100	0	0	100	0	0	100	0	0
	WAG-C10	100	0	0	100	0	0	100	0	0	100	0	0
	MLHe	100	0	0	100	0	0	100	0	0	100	0	0
1	CAT-GTR	100	0	0	100	0	0	100	0	0	100	0	0
	MLH	100	0	0	58	34	8	0	0	100	100	0	0
	GTR	100	0	0	86	10	4	0	0	100	100	0	0
	WAG-C10	100	0	0	52	38	10	18	0	82	100	0	0
	MLHe	100	0	0	50	38	12	94	0	6	98	2	0
5	CAT-GTR	100	0	0	86	10	4	98	0	2	94	2	4

	MLH	98	2	0	48	48	4	0	0	100	100	0	0
	GTR	96	0	4	82	6	12	0	0	100	100	0	0
	WAG-C10	96	0	4	52	44	4	2	0	98	100	0	0
	MLHe	98	0	2	54	42	4	72	0	28	94	6	0
10	CAT-GTR	96	2	2	82	17	1	80	2	18	92	8	0

Table Legend: The table summarizes the Recovery Rates (the frequency with which the target (i.e. true) tree is recovered and the frequency with which unresolved (U) and incorrect (Wrong; W) trees are recovered, for the large datasets. BLMF is the Branch Lengths Multiplier Factor (see Methods, Main text and Fig. 1). Results are reported for all experimental conditions tested. We consider a tree to be unresolved when support (either posterior probability of ultrafast bootstrap) is lower than 50%, irrespective of whether the topology of the recovered tree is correct or not (see Fig. 2). All the data summarised in this table can be found in Allresults.xls (https://figshare.com/s/70fc81480557472bd970).

Table S3: The accuracy of different models with the 5-gene heterogeneous alignments simulated in Phylobayes

	Tree		
	Felsenstein		
Model	RR	U	W
MLHo	49	3	48

GTR	45	1	55
Partition	38	2	60
LG-C10	78	1	21
MLHe	90	0	10
CAT-GTR	96	0	4

Table Legend: The table summarizes the Recovery Rates (the frequency with which the target (i.e. true) tree is recovered and the frequency with which unresolved (U) and incorrect (Wrong; W) trees are recovered, for the 5-gene heterogeneous datasets simulated in Phylobayes (BLMF= 1 – see Methods). These analyses were explicitly performed to compare Partitioned Models against the other models tested in this study. Results are reported for all experimental conditions tested. We consider a tree to be unresolved when support (either posterior probability of ultrafast bootstrap) is lower than 50%, irrespective of whether the topology of the recovered tree is correct or not (see Fig. 3).

Table S4: Performance of all models averaged across all experimental conditions

Simulation	Data type	MLH		GTR		Partition		WAG-C10		MLHe		CAT-GTR	
		P	U	P	U	P	U	P	U	P	U	P	U
	All	0.65	0.07	0.68	0.03	NA	NA	0.69	0.07	0.79	0.11	0.85	0.05
Elynx	Hetero	0.5	0	0.5	0.01	NA	NA	0.57	0.01	0.81	0.04	0.83	0.04

	Homo	0.8	0.14	0.86	0.06	NA	NA	0.8	0.14	0.78	0.17	0.87	0.06

Table Legend: This table summarizes the Performance of all the models considered in this study (see also Fig. 4). All: Performance and uncertainty of every model averaged across all experimental conditions and datasets (Farris and Felsenstein trees; BLMF= 0.1, 1, 5, 10; and both the homogeneous and heterogeneous data, and dataset sizes). Hetero: as above but calculated over the heterogeneous data only. Homo: as above but calculated over the homogeneous datasets only. Elynx: Elynx simulated datasets. Performance (P)= (RR-W). U (as in Table S1-S3 and Figs 2 and 3) is the proportion of trees (either correctly or incorrectly resolved) that had support lower than 50% (either ultrafast bootstrap or posterior probability - methods for details) averaged over all considered experimental conditions. All the data summarised in this table can be found in Allresults.xls (https://figshare.com/s/70fc81480557472bd970).

Table S5: Estimates of key branch lengths on the Farris tree (Elynx simulated datasets)

		BLMF					
Model	Clade	1			10		
		Target branch length	Homogeneous data	Heterogeneous data	Target branch length	Homogeneous data	Heterogeneous data
MLHo	ABEF	0.0182	0.0183	0.0372	0.0182	0.0321	0.3053
	EF	1.0319	1.0326	0.658	10.3195	10.2263	4.17
	AB	0.2462	0.2458	0.1677	2.46194	2.4459	1.0026
GTR	ABEF	0.0182	0.0181	0.0297	0.0182	0.0473	0.3302
	EF	1.0319	1.0384	0.7775	10.3195	10.2322	4.6189
	AB	0.2462	0.2468	0.1897	2.46194	2.4348	1.0819
WAG-C10	ABEF	0.0182	0.0176	0.0252	0.0182	0.0397	0.2324
	EF	1.0319	1.0447	0.8461	10.3195	9.9984	6.508
	AB	0.2462	0.248	0.2022	2.46194	2.4285	1.5151
MLHe	ABEF	0.0182	0.0188	0.0383	0.0182	0.0387	0.1111
	EF	1.0319	1.0311	0.7071	10.3195	10	8.492
	AB	0.2462	0.2456	0.1792	2.46194	2.4375	1.9741
CAT-GTR	ABEF	0.0182	0.0187	0.0218	0.0182	0.0468	0.0807
	EF	1.0319	1.0326	1.019	10.3195	10.251	10.0762
	AB	0.2462	0.2459	0.2431	2.46194	2.4384	2.4092

Table Legend: The table report estimated branch lengths for the long-branched clades in the Farris tree (AB) and (EF), and for the short branch subtending, the clade composed by the two long branched lineages (ABEF). The table shows that when the data are homogeneous and the long branches have not been extended to exacerbate attraction artefacts all models perform well. When the data are heterogeneous MLHe and CAT-GTR perform better, particularly when the length of the branches has been elongated 10 times to exacerbate attraction artifacts. However, we note that all models tend to overestimate the length of ABEF, indicating that while available heterogeneous models are significantly less affected by LBA than homogeneous ones, no available model is completely immune to LBA providing an explanation for our observation that when branch lengths are exacerbated (10 times) all models find it difficult to identify the Felsenstein tree (even though heterogeneous models perform better) while at the same time all models have a good RR for the Farris tree in the same conditions (see Fig. 5). All the data summarised in this table can be found in Allresults.xls (https://figshare.com/s/70fc81480557472bd970).

Table S6: number of site frequency categories used by CAT-GTR analyses

		Datasets			
		Small		Large	
	Data type	Heterogeneous	Homogeneous	Heterogeneous	Homogeneous
Number of Categories	Mean	83.05	1.185	123.4824	1.819444
	Mode	87	1	122	1
	Max	119	4	162	8
	Min	51	1	96	1

Table Legend: Number of site frequency categories used by CAT-GTR when analysing homogeneous and heterogeneous datasets simulated using Elynx. Values are obtained from Phylobayes trace files (Nmode parameter), after having excluded 5,000 cycles (burin). All datasets simulated under the Farris and Felsenstein trees (BLMF=1) were considered (see Fig. 6). All the data summarised in this table can be found in Allresults.xls (https://figshare.com/s/70fc81480557472bd970).
