# Supplementary figures and images for: Phylogenomic mixture models outperform homogeneous and partitioned models
Source: Mol Biol Evol. 2026 Apr 9;43(5):msag090. doi: 10.1093/molbev/msag090 (PMC13197666; doi:10.1093/molbev/msag090)

Figure S1

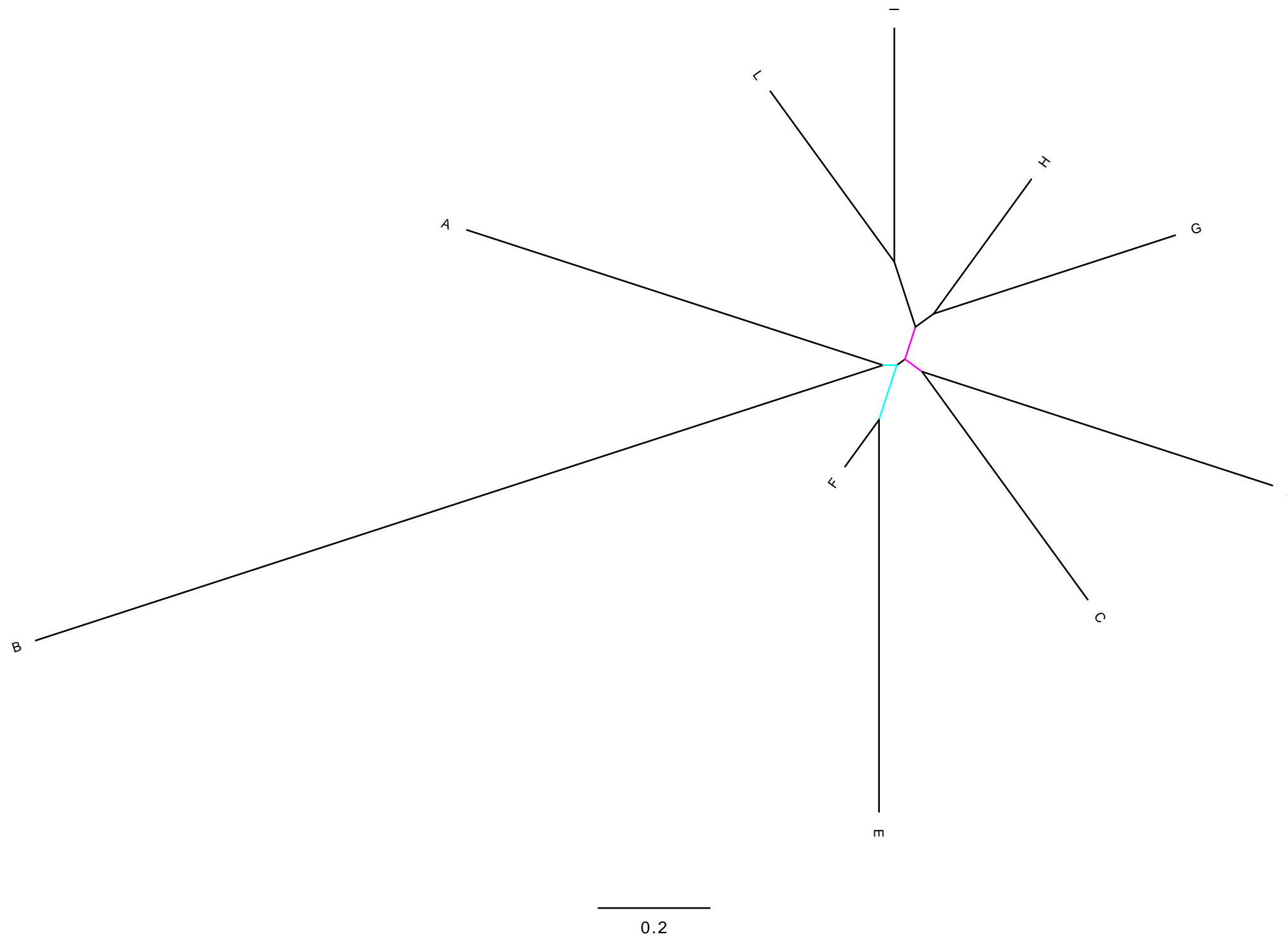

Supplement: msag090_Supplementary_Data [file msag090_supplementary_data.zip › FIGS1.pdf]

Fig. S2

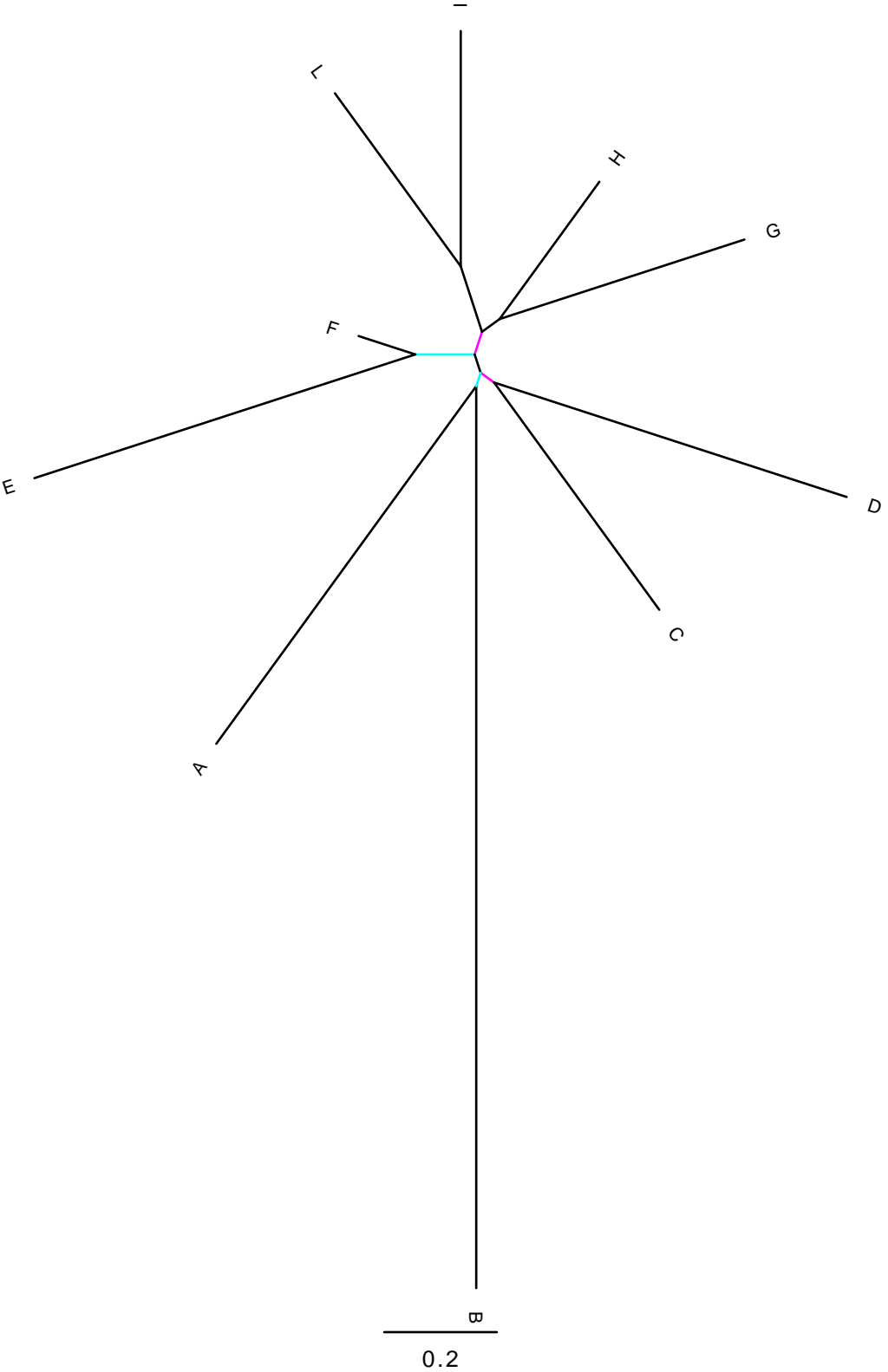

Supplement: msag090_Supplementary_Data [file msag090_supplementary_data.zip › FIGS2.pdf]

Fig. S3

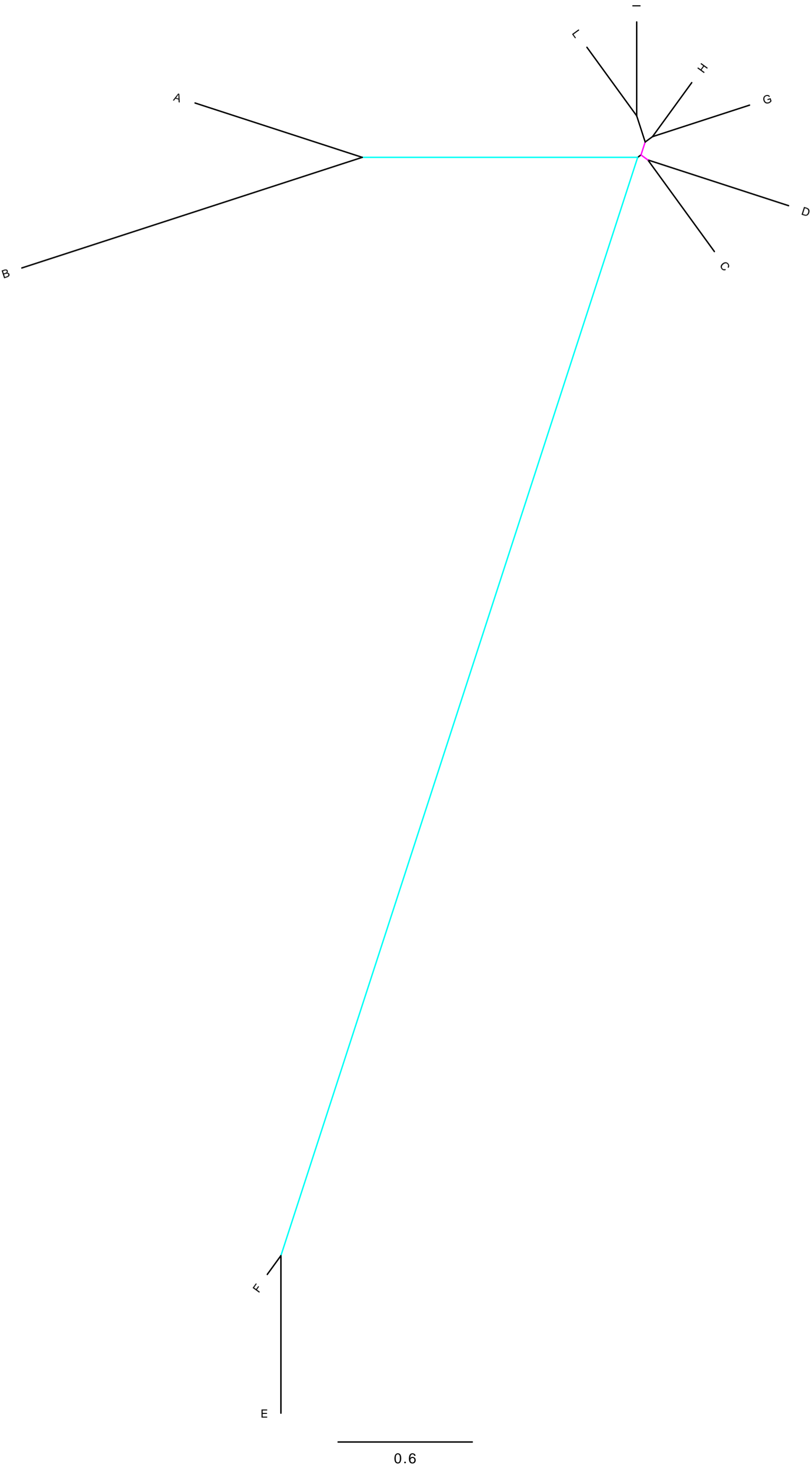

Supplement: msag090_Supplementary_Data [file msag090_supplementary_data.zip › FIGS3.pdf]

Fig. S4

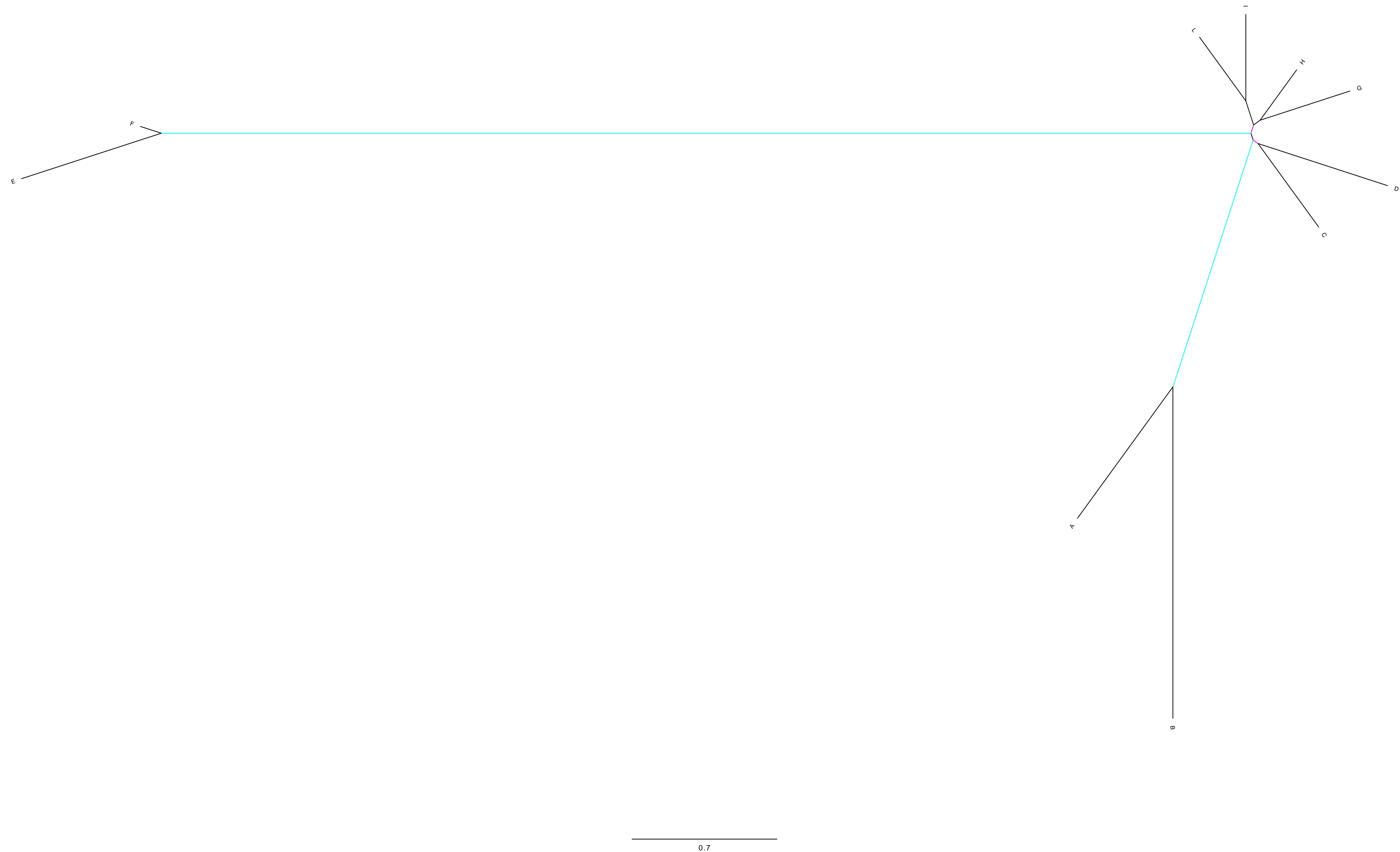

Supplement: msag090_Supplementary_Data [file msag090_supplementary_data.zip › FIGS4.pdf]

Fig. S5

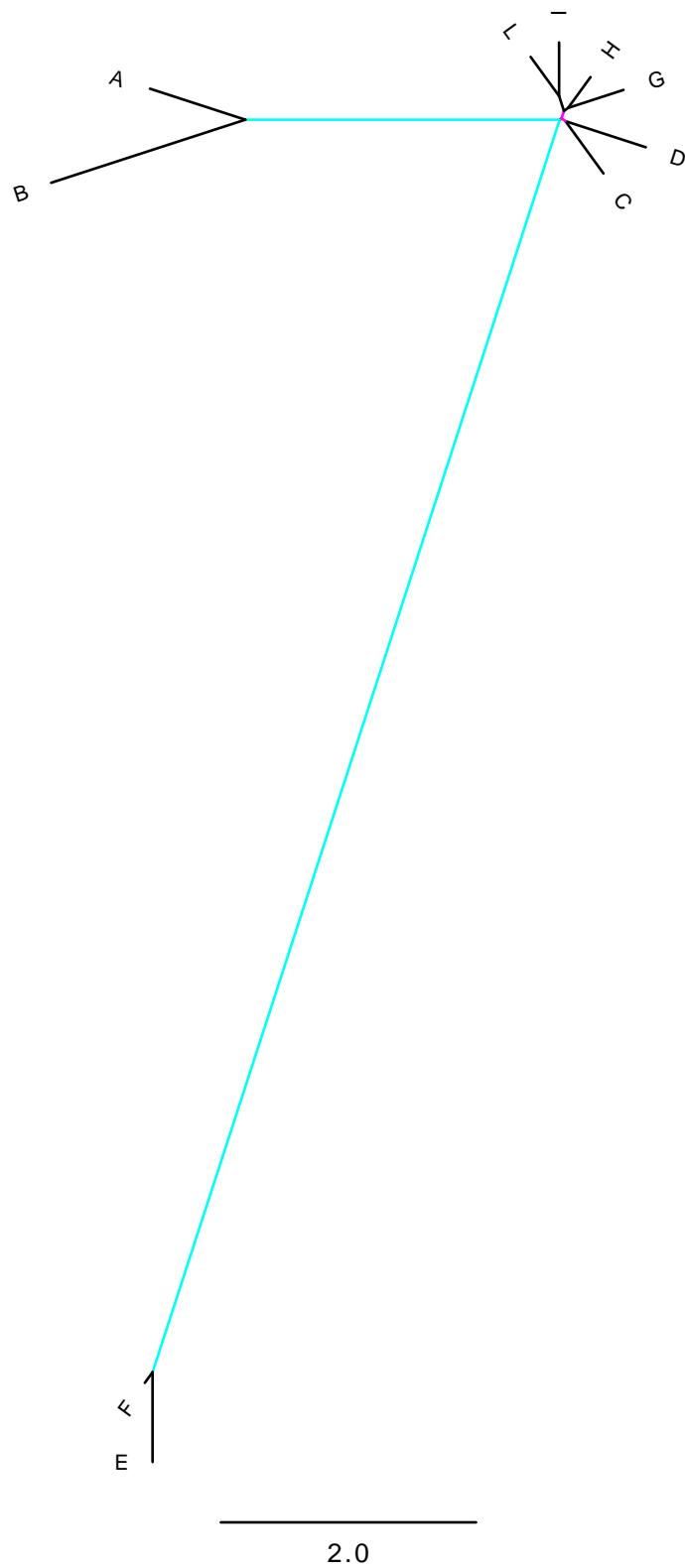

Supplement: msag090_Supplementary_Data [file msag090_supplementary_data.zip › FigS5.pdf]
